# Genomic Survey of LRR-RLK Genes in *Eriobotrya japonica* and Their Expression Patterns Responding to Environmental Stresses

**DOI:** 10.3390/plants13172387

**Published:** 2024-08-27

**Authors:** Mengqi Yang, Tian Min, Teja Manda, Liming Yang, Delight Hwarari

**Affiliations:** State Key Laboratory of Tree Genetics and Breeding, College of Biology and the Environment, Nanjing Forestry University, Nanjing 210037, China; yangmengqi0908@163.com (M.Y.); tmin@njfu.edu.cn (T.M.); teja.manda27@gmail.com (T.M.)

**Keywords:** *Eriobotrya japonica*, *LRR-RLK* genes, abiotic stress response, gene evolution, phylogeny

## Abstract

The impact of global warming is increasing and thus exacerbating environmental stresses that affect plant yield and distribution, including the *Eriobotrya japonica* Lindl (Loquat tree). *Eriobotrya japonica*, a member of the Rosaceae family, is valued not only for its nutritious fruit but also for its medicinal purposes, landscape uses, and other pharmacological benefits. Nonetheless, the productivity of *Eriobotrya japonica* has raised a lot of concern in the wake of adverse environmental conditions. Understanding the characteristics of the *LRR-RLK* gene family in loquat is crucial, as these genes play vital roles in plant stress responses. In this study, 283 *LRR-RLK* genes were identified in the genome of *E. japonica* that were randomly positioned on 17 chromosomes and 24 contigs. The 283 EjLRR-RLK proteins clustered into 21 classes and subclasses in the phylogenetic analysis based on domain and protein arrangements. Further explorations in the promoter regions of the *EjLRR-RLK* genes showed an abundance of cis-regulatory elements that functioned in growth and development, phytohormone, and biotic and abiotic responses. Most cis-elements were present in the biotic and abiotic responses suggesting that the *EjLRR-RLK* genes are invested in regulating both biotic and abiotic stresses. Additional investigations into the responses of *EjLRR-RLK* genes to abiotic stress using the RT-qPCR revealed that *EjLRR-RLK* genes respond to abiotic stress, especially heat and salt stresses. Particularly, *EjapXI-1.6* and *EjapI-2.5* exhibited constant upregulation in all stresses analyzed, indicating that these may take an active role in regulating abiotic stresses. Our findings suggest the pivotal functions of *EjLRR-RLK* genes although additional research is still required. This research aims to provide useful information relating to the characterization of *EjLRR-RLK* genes and their responses to environmental stresses, establishing a concrete base for the following research.

## 1. Introduction

*Eriobotrya japonica* Lindl. (Rosaceae) is an evergreen shrub or small tree, with a rounded crown, short trunk, and woody twigs, and is native to southern China. Normally referred to as the Loquat tree, it is famous as a Chinese medicine for soothing sore throats and coughs; its other benefits include fruits and landscape [1], natural dye and bio-mordant [2], and other pharmacological benefits [3,4]. However, the growth, development, and yield of the Loquat tree is often severely affected by abiotic stresses such as low temperature, high temperature, and drought and is on the verge of extinction due to global climate change [5] and other environmental stresses [6]. 

The *Leucine-rich repeat-like kinases* (*LRR-RLKs*) are critical for plant growth and development and also play a crucial role in plant adaptation to environmental stresses [7]. They form one of the largest gene families in plants [8]. Their biochemical structures consist mainly of three functional domains: an extracellular domain (ECD), an intracellular kinase domain (KD), and a transmembrane domain [9]. The extracellular leucine-rich repeat (LRR) domains are involved in protein–protein interactions, and intracellular serine/threonine kinase domains mediate signal transductions [9,10]. Furthermore, the large expands of LRR repeats in the LRR-RLK ECD perceive a wide range of ligands, such as minute compounds, peptides, and other proteins [11]. The intracellular kinase domain (KD) observable in protein kinases contains 12 conserved subdomains that depict identical core two-lobed catalytic structures in three dimensions (3D) [12,13]. In addition, LRR-RLK categorization is mostly based on the phylogeny of KDs, classifying plant *LRR-RLK* genes into 15–29 subgroups [14,15]. Several subclasses have been shown to share a rich conserved expanse of leucine denoted by a LxxLxLxNxL(s/t)GxLPxxLxx, with L denoting the hydrophobic amino acid, N for asparagine, threonine, serine, or cysteine, and x representing any variable residue [16]. Additionally, the ‘L(s/t)GxLP’ region has been shown to shape into a plant-specific β-strand that detects the conformation of the LRR stacks into super-helical structures. The ‘LxxLxxN’ region was also shown to model a curved parallel sheet lining within the edge of their solenoid [17]. 

Given the increase in the prevalence of climate change and abiotic stressors, understanding the function of *LRR-RLK* genes in response to stresses has become important. *LRR-RLK* genes mediated drought responses by modulating stomatal closures, root architecture, and the expression of drought-responsive genes [18,19]. For example, the *ERECTA* in *A. thaliana* has been shown to regulate stomatal density size [20]. *LRR-RLK* genes have been implicated in cold stress responses by modulating cold acclimation processes and triggering the expression of cold-responsive genes. Additional investigations exhibited that *LRR-RLKs* modulated the production of reactive oxygen species (ROS) and stabilized the cellular structure, thereby maintaining metabolic functions [21,22]. Heat stress poses a significant threat to plants, reducing yields and quality. The *LRR-RLK* genes modulated heat shock proteins (HSPs) and other mechanisms that maintained protein stability and prevented cell damage under high temperatures [23]. In salt stress, the *LRR-RLKs* regulated ion transporters, osmolyte production, and salt stress-responsive gene expression [24]. 

Although the *LRR-RLKs* are significant for plant adaptation to environmental stresses, they pose several limitations. One major limitation is their functional redundancy, making it complex to understand the precise function of individual genes [21]. Another is potential pleiotropic effects, where their overexpression or silencing may lead to unplanned results on plant growth and development [7]. For instance, certain *LRR-RLKs* may enhance salt stress while at the same time reducing yield. Nonetheless, advancements in plant biochemistry and molecular biology provide endless prospects for *LRR-RLKs* in crop improvements. Fields of study in transcriptomics and gene editing, CRISP/Cas 9, may offer ways to overcome these limitations. 

Over time, research has identified and elucidated the *LRR-RLK* gene family in several plants and revealed their functions in the regulation of environmental stress, including heat, cold, drought, salt, and nutrient treatment [21,25]. To mention a few, *LRR-RLK* genes have been identified in several plants, including Arabidopsis [26], *Zea mays* [27], rosacea plants [28], Saccharum [29], and Gossypium species [30]. However, the *LRR-RLK* gene family has not been identified in the *Eriobotrya japonica.* In this research, we identified 283 *LRR-RLK* genes in *Eriobotrya japonica* that were located on 17 chromosomes and several contigs. We performed several analyses, including evolution, domain conservation and arrangements, and gene expression responses to gain insight into their function roles. Thus, we concluded that this research provided valuable gene-based information for additional molecular breeding programs to improve plant abiotic tolerances.

## 2. Results

### 2.1. Genome-Wide Identification and Classification of the LRR-RLK Genes in Eriobotrya japonica

**Figure 1:**
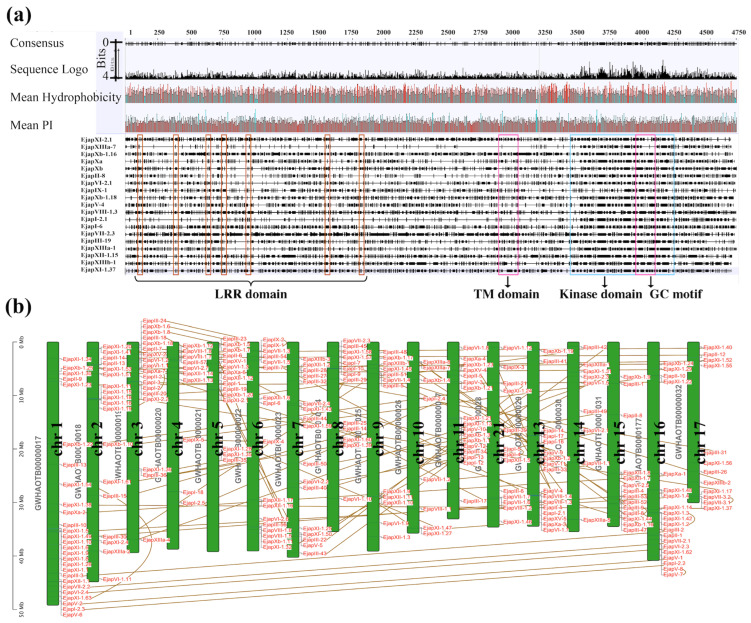
Characterization of *EjLRR-RLK* genes. (**a**) Depicts the multiple sequence alignments of EjLRR-RLK proteins as generated by Geneious Prime, showing typical conserved motifs and domains labeled in black. LRR domain: conserved LRR repeats region, TM domain: transmembrane domain and the GC motif is marked yellow within the kinase domain. The isoelectric point (pI) and the hydrophobicity graphs are shown above the image of multiple sequence alignments marked in red. (**b**) The chromosomal locations and their respective gene collinearities. Each chromosome is marked in black, and all genes are marked in red. Their respective location and sizes are shown by a far-left scale.

To gain insight into the distribution of the *LRR-RLK* genes in *Eriobotrya japonica*, we mined for protein kinases (PKs) in the *E. japonica* genome resources. We identified 1829 typical protein kinases (Supplementary File S1); after removing redundant and overlapping sequences, 283 EjLRR-RLK protein sequences were obtained. The 283 EjLRR-RLK proteins carried typical conserved domains, an extracellular domain (ECD), a transmembrane domain, and an intracellular kinase domain (KD) (Figure 1a; Supplementary Figure S3). In addition, the obtained EjLRR-RLK ECD were branded with varying LRR repeats. Then, the 283 EjLRR-RLKs were classified and renamed into 12 classes and 9 subclasses labeled in Roman numeral (I-XIII) based on previous publications in Arabidopsis and rice [7,17]. Our findings showed that Class IV and VIV in *A. thaliana* were absent in *E. japonica.* Furthermore, classes I, VI, X, and XI had additional classifications of two subclasses, VII had three subclasses, and the rest had no additional classifications (Supplementary File S2). Subclasses XI-1 and III were the core subclasses with 63 and 57 members, respectively. While the other subclasses contained at most 25 members and at least 2 members. In addition, protein lengths varied based on group classifications (Supplementary File S2), subfamily XI-1 had the longest protein sequences ranging from 874 to 1257 amino acids (aas), and that of subfamily Xb-1 ranged from as low as 363 aas. The physicochemical analyses showed an isoelectric point (pI) range of 4.5 to 6.7, and a subcellular localization in the plasma membrane Supplementary File S2.

In addition, the TBtools software v 2.112 was used to determine the gene location on each chromosome (Figure 1b; Supplementary File S2). All the *EjLRR-RLKs* were located on 17 chromosomes and 24 contigs, with each chromosome carrying at least 27 genes. Additional information relating to *EjLRR-RLKs* is available in (Supplementary File S2).

### 2.2. Gene Structure and Conserved Domain Analyses 

**Figure 2:**
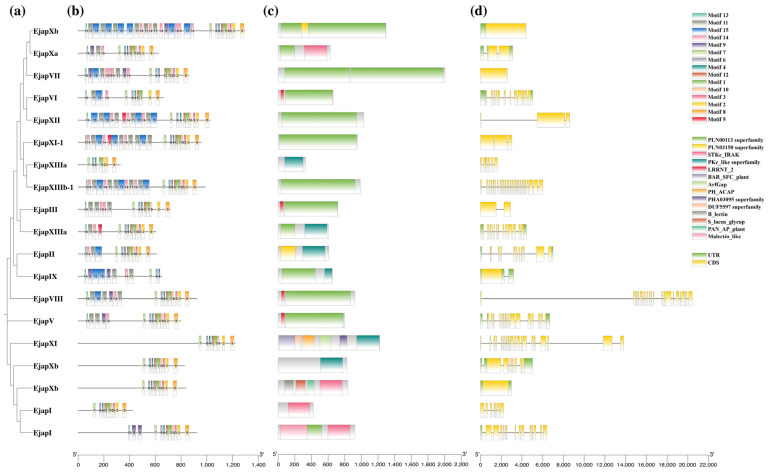
Gene evolution and domain conservation in *EjLRR-RLK* genes. (**a**) The phylogenetic relationships of LRR-RLKs as generated using the Mega software v 11.0.13. Representative EjLRR-RLK proteins were used for the evolutionary analysis. (**b**) The motifs in the LRR-RLK proteins, shown in different colors, as described in the top right corner key. (**c**) Conserved domain analysis, each conserved domain is depicted in a different color as defined in the key second from the top. (**d**) Exon–intron arrangements are shown in green for UTR (intron) and yellow for CDS (exon).

Gene structure prediction provides substantial insight into a gene family’s evolution [31]. Exon and intron numbers of the obtained *EjLRR-RLKs* varied and suggested clustering of the 283 genes into identical classes based on their variations (Supplementary Figure S1; Figure 2a–d). Classes Xb-2, Xa-1, VII-2, XII, XI-1, III, IX, and Xb-2 had between one and two exons and at most two introns at the sequence terminals. The remaining classes had more than three exons; surprisingly, family XIIIb was without introns and with more than three exons. Interestingly, different gene classes had different exon structures; those with fewer exons had elongated exons, and most gene classes had intron structures that were nearly the same size, except Xb-3, which had an elongated intron flanking its C-terminal. The PKinase and LRR domains commonly make up the fundamental structure of the LRR-RLK gene [7]. Analysis in the LRR-RLK conserved domains showed that protein domain compositions of distinct subclasses differ (Figure 2c). The Pkinase and LRRNT_2 (leucine repeat N-terminal) were present in most subclasses (Figure 2b; Supplementary File S1, Supplementary Figure S1). Additionally, some subclasses, including XI, II, and XIII had a combined Pkinase and Pkinase_Tyr domains with a maximum of six conserved domains, and subfamily XI possessed the largest number of conserved domains.

### 2.3. Phylogenetic Analysis of the LRR-RLK Gene Family

**Figure 3:**
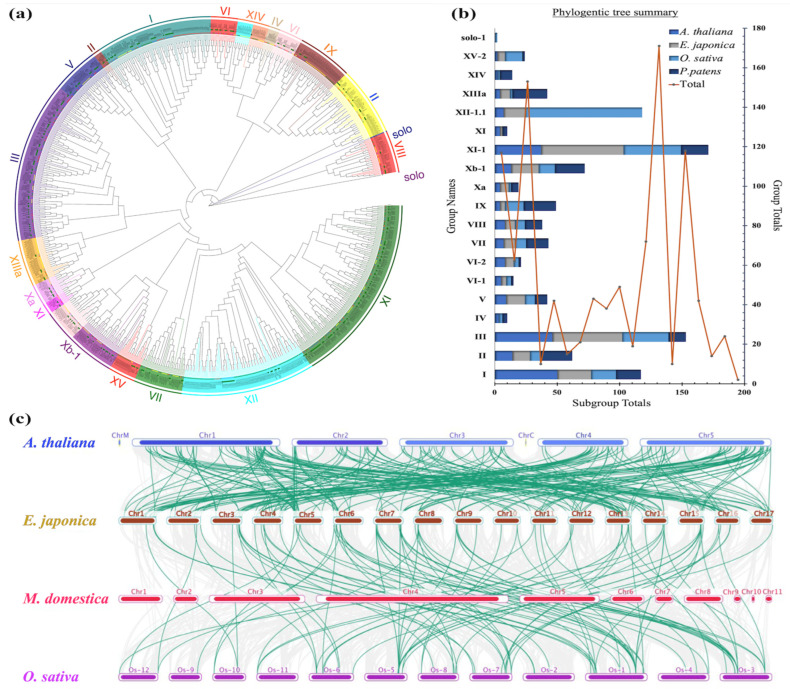
Evolution analysis of the LRR-RLK genes in *E. japonica.* (**a**) The phylogenetic relationship of 1032 LRR-RLK proteins. Different color schemes show each of the groups denoted as I–XV. (**b**) Summary of the LRR-RLK total numbers in (**a**). (**c**) The interspecies *LRR-RLK* gene collinearity between *E. japonica*, *A. thaliana*, *O. sativa*, and *M. domestica*. Green curvy lines show collinearity and chromosomes are labelled above.

Gene constructions and functionality are facilitated by the systematic classifications of a gene family controlled by protein phylogenetics. In this study, a phylogenetic tree was constructed using the MEGA X software using the neighbor-joining tree (NJT) method (Figure 3a). In total, 1032 LRR-RLK full protein sequences from four plants, *A. thaliana*, *E. japonica*, *O. sativa*, and *P. patens*, were grouped into several classes and subclasses by earlier studies [28,30]. In detail, the LRR-RLK proteins showed a diversification that formed 5 branches, 21 cluster groups that made up 15 classes, and 7 subclasses. This finding also showed that all these cluster groups had fanned out from a common ancestral LRR-RLK protein. This finding implies that the LRR-RLKs diverged through speciation mostly from a single ancestral protein, probably via adaption and other evolutionary strategies. A more thorough examination revealed that two main branches carried subfamily VIII-1 and family II and a solo rice LRR-RLK protein. In addition, classes XI-1 and III were the largest clusters with 340 and 146 protein sequences, respectively (Figure 3b). Also, it can be inferred that protein sequences clustered in identical classes or subclasses may exhibit similar protein domains, structures, and possible functions. In this research, LRR-RLK proteins from *E. japonica* were randomly distributed in all the cluster groups except in the classes XIV and IV. This may suggest that EjLRR-RLK clustered with other proteins in similar cluster groups have identical characteristics. Notably, some EjLRR-RLK proteins clustered in similar classes with the *P. patens* showing that they carry evolutionarily conserved domains. 

### 2.4. Gene Duplication, Gene Collinearity, and Ratio of the Number of Nonsynonymous Substitutions per Nonsynonymous Site (Ka) to the Number of Synonymous Substitutions per Synonymous Site (Ks)

**Figure 4:**
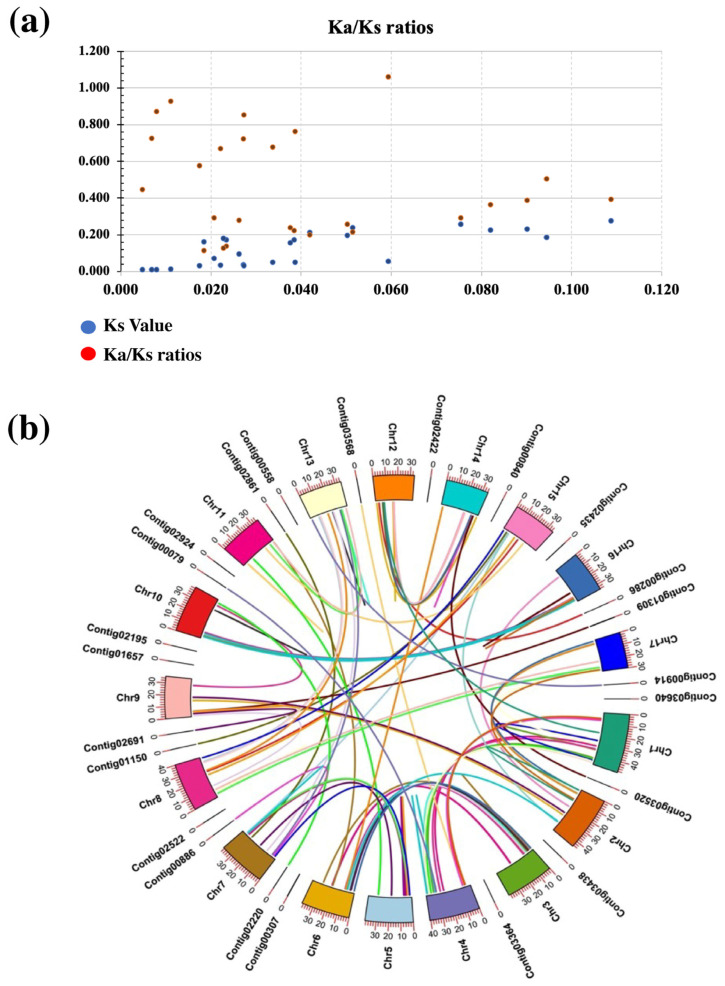
The synonymous and nonsynonymous values of identified *EjLRR-RLKs.* (**a**) The Ka, Ks-values, and their respective Ka/ks ratios. Dots in the graph are defined in the key below. (**b**) The synteny analysis in *E. japonica* genes. Each chromosome is labeled outside and a colored curvy line linking individual chromosomes represents linked genes. The sizes of each chromosome are shown outside graduated chromosomes.

The collinearity analysis showed that *E. japonica* had more orthologous genes with *A. thalina* as compared to *M. domestica* and *O. sativa* (Figure 3c). Indicating that *E. japonica* might be evolutionary closer to *A. thaliana* than *M. domestica* and *O. sativa*. Gene duplication poses an impact on the expansion of certain plant gene classes [32]. Two key gene duplication events have been shown to significantly contribute to this expansion, namely segmental and tandem duplication events [33]. Selection pressure on particular DNA or protein is evaluated by the synonymous and nonsynonymous values and their ratios [34]. In this research, we calculated these values to gain insight into the origin of the duplicated genes (Figure 4a; Supplementary File S3). We observed 106 connected genes, all but *EjXIIIa-7* linked to *EjXIIIa-8* had Ka/Ks ratios less than 1 (Ka/Ks < 1), showing a purifying selection. This result is evidence that the *EjLRR-RLK* genes are crucial for adaptation of the *E. japonica* to environmental stresses. 

To understand the method of gene expansion in the *EjLRR-RLK* gene family, we compared the tandem and segmental duplications in *E. japonica* LRR-RLK genes using the synteny analysis (Figure 4b). Results showed 18 tandem arrays, contributing 59% (18/104) of the duplicated genes. Most of the tandem duplication were present in the XI family contributing 27.8% (5/18) of the total tandem arrays. Segmental duplication is another source of gene family enlargement; it upsurges gene and genome densities [35]. We obtained 88 segmental duplications that contributed 83% (88/106) toward the expansion of the *EjLRR-RLK* gene family (Figure 4b). Most segmental duplications were present in III and XI-1 classes contributing 26% (23/88) and 23% (20/88) of the total segmental duplications. Similar results were reported in the gene duplication studies of other Rosaceae genomes [28].

### 2.5. Putative cis-Regulatory Elements

**Figure 5:**
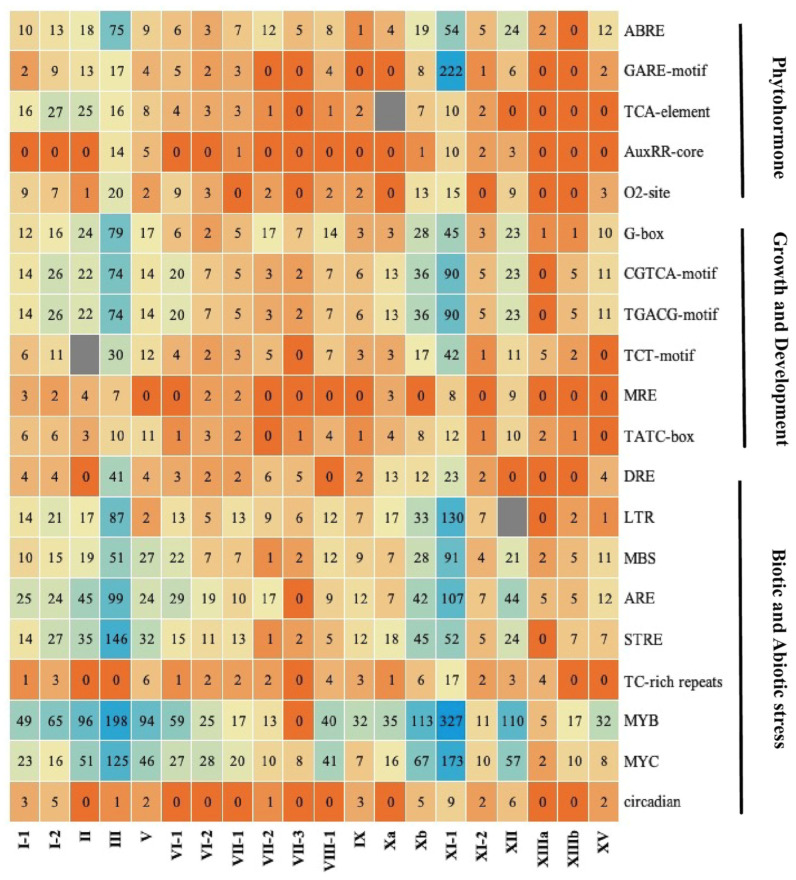
Cis-regulatory elements of *EjLRR-RLKs*. Total numbers of cis-regulatory elements identified in *E. japonica LRR-RLK* promoter regions. LRR-RLK genes are denoted in the x-axis, while the boxes show varying numbers. Different color backgrounds reflect different ranges.

Comprehension of gene transcriptional regulation and function entails an evaluation of the cis-regulatory elements found in the promoter region [36]. In this research, a 2 kb region of the discovered *EjLRR-RLK* genes was considered as a potential promoter region. Using the online Plant Care Database, 6281 cis-regulatory elements were identified and categorized into three response factors, growth and development, hormonal responses, and biotic and abiotic responses. (Supplementary Figure S2; Supplementary File S4; Figure 5). Interestingly, their distributions and compositions varied with individual genes. However, we observed that within the same classes and subclasses, the cis-element distributions exhibited a relatively similar trend. Comparisons of the various response components revealed that two of them, growth and development, and biotic and abiotic responses accounted for 22% and 64% of the total elements. Furthermore, of all the response factors examined, classes III and XI-1 had the most cis-elements, indicating that these classes have functional roles related to growth and development as well as biotic and abiotic responses. Particularly, *EjXI-1.30*, *EjXI-1.5*, and *EjI-3* were rich in biotic and abiotic responses, including ABRE, GARE-motif, DRE, and the LTR. This finding suggests that these genes are critical in drought, cold, heat, and salt stress responses (Supplementary Figure S2). Wholly, this finding implies that the *EjLRR-RLK* genes are responsive to diverse stimuli, including abiotic stresses. 

### 2.6. Protein–Protein Interaction and 3D-Protein Anlyses 

**Figure 6:**
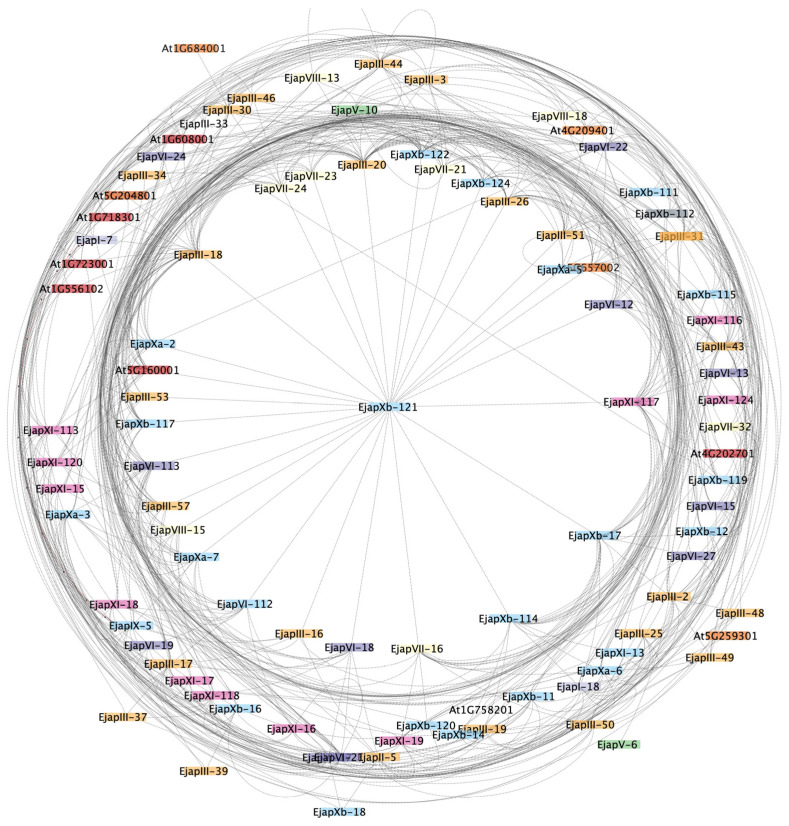
Protein interaction network of *EjLRR-RLKs*. Different color schemes show different EjLRR-RLK groups.

Protein–protein interactions (PPIs) are essential in defining protein functions and the effect of protein absence or presence. We used the online String database to examine the protein interactions among different LRR-RLK proteins (Figure 6). The findings revealed that EjLRR-RLK protein classes intricately link to perform different functional roles. Hence, we concluded that interactions among EjLRR-RLK protein classes facilitate plant responses to environmental stress and their respective regulations. We observed that the protein families were densely interconnected. Several LRR-RLK groups interacted with each other probably for efficient role functions. This finding suggested that the LRR-RLK gene family in *E. japonica* interact to perform their biological roles. In detail, most of the proteins were linked to EjapXB-12.1 of group II, suggesting a possibility that this gene may act as a control hub mediating several protein functions [17]. 

**Figure 7:**
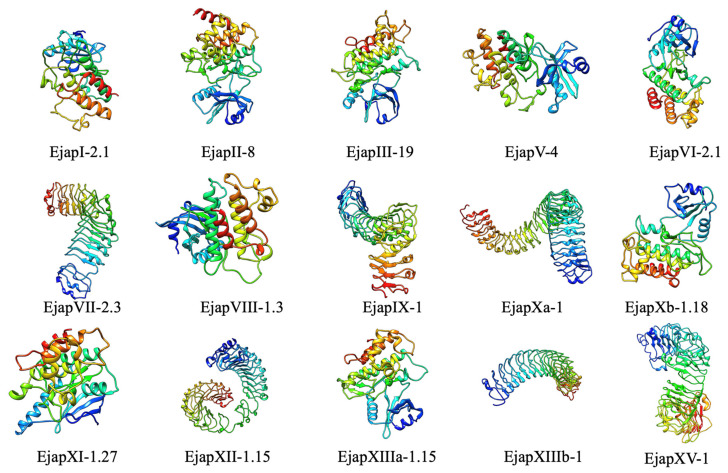
Three-dimensional protein structures of EjLRR-RLKs representatives from each subfamily.

On the other hand, research has shown that LRR repeats present in a protein are responsible for protein stacking into super-helical forms [29]. In this study, we used the online SWISS-model tool to examine the structures of 15 EjLRR-RLK proteins. Generally, the hydrophobic regions of the EjLRR-RLKs were buried in inner superhelices created by multiple LRRs. Furthermore, the variable residues of the LRR backbone exhibited lower hydrophilicity than anticipated to support correct protein folding, while the conserved residues were more hydrophobic than the variable residues. In detail, the EjLRR-RLKs from various classes showed distinct protein structures; however, those from classes VII, IX, X, and XII exhibited relatively different structures (Figure 7). 

### 2.7. Gene Ontology (GO) Analysis of EjLRR-RLK Genes 

**Figure 8:**
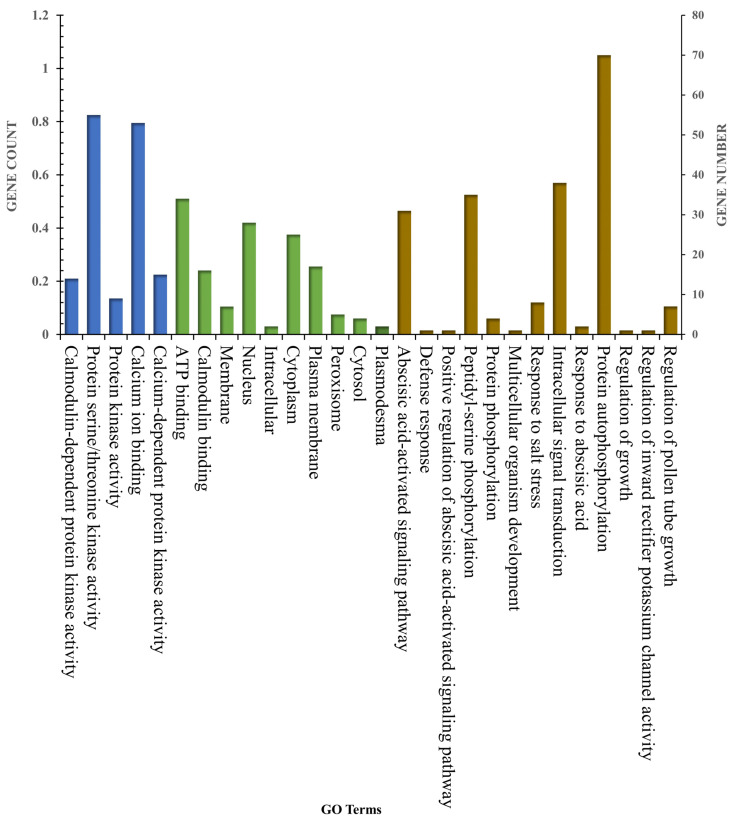
Gene ontology analysis in *E. japonica LRR-RLKs*, categorized by biological processes (shaded in blue), molecular functions (shaded in brown), and cellular components (shaded in green). The chart highlights the predominant roles of these genes in processes such as signal transduction, receptor activity, and membrane localization, reflecting their roles in plant immunity and plant growth.

To predict the possible roles of the identified *EjLRR-RLK* genes, the Gene Ontology Annotations were retrieved and grouped into three groups, including cellular components (CC), molecular function (MF), and biological processes (BP) (Figure 8). In particular, the molecular function category had the largest number of genes present. All the *EjLRR-RLKs* searched were involved in ATP binding, protein binding, and DNA binding. This suggests that *EjLRR-RLK* genes are well invested in binding. However, other single genes were involved in GTPase activator activity and transmembrane receptor protein tyrosine kinase function. In the CC category, genes were present in the nucleus and cytoplasm separately, affirming previous findings that LRR-RLKs are localized in the cytoplasm and nucleus (Figure 8). In the BP processes, all the *EjLRR-RLK* genes were involved in protein phosphorylation and two were involved in DNA-templated transcription and pollen recognition separately (Figure 8). Wholly, this finding suggests that the *EjLRR-RLK* gene family functions are diverse and play diverse roles in *E. japonica.*

### 2.8. RT-qPCR Expression Patterns of EjLRR-RLK Genes

**Figure 9:**
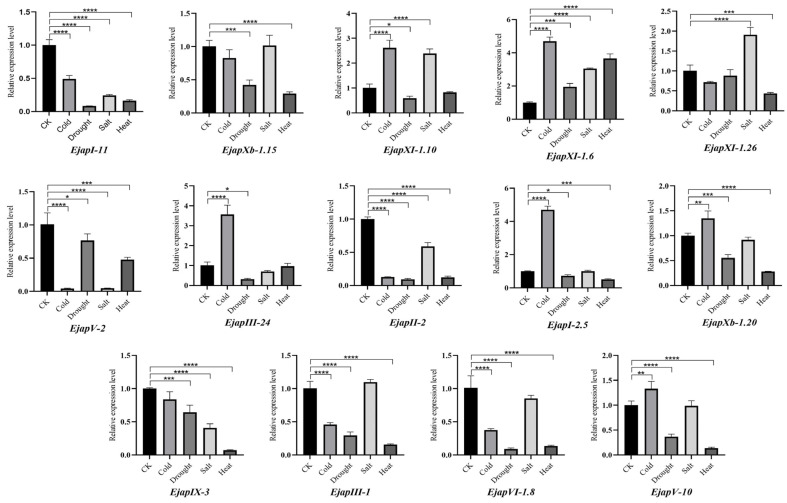
The relative expression patterns of 14 *EjLRR-RLK* genes in different abiotic stresses, cold, drought, salt, and heat, compared to control check (CK) using the RT-qPCR. The values were illustrated as bars using Graph Pad Prism. The asterisk (*) above the bars shows significant differences. The statistical significance of the differences was validated by Dunnett’s multiple comparison test (* *p* < 0.05, ** *p* < 0.01, *** *p* < 0.001, **** *p* < 0.0001).

The RT-qPCR analysis measured the relative expression patterns of 14 *EjLRR-RLK* genes in the leaves (Figure 9). The *EjLRR-RLK* genes were selected based on the cis-regulatory elements and the expression values of their homologs in other publications. Generally, most of the *EjLRR-RLK* genes had higher expression values than the control check (CK). In detail, at least 50% of the investigated genes were highly upregulated in the cold stress. Notably, *EjapXI-1.6* and EjapI*-2.5* were found to be above 4-fold higher than the control sample, while *EjapXI-10*, *EjapXb*, and *EjapI-2.5* had a fold change two times that of the control. Similar results were observed in the salt stress; most of the *EjLRR-RLK* genes had a higher fold change. The expression values of *EjapXI-1.10* and *EjapIII-1* had a fold change above two. Wholly, we observed higher expression values of the selected *EjLRR-RLK* genes in cold and drought stress. Therefore, it can be summarized that *EjLRR-RLK* genes are involved in abiotic stress responses and different gene classes exhibit different expression patterns suggesting that different *EjLRR-RLK* genes regulate abiotic stresses differently.

## 3. Discussion

The *LRR-RLK* genes form one of the largest gene classes in plants; they are involved in critical plant roles, including plant growth and development, and stress responses [7]. Several pieces of research have already identified the *LRR-RLK* genes in numerous plant species such as citrus species, maize, *Populus trichocarpa*, Rosaceae species, and others [26,27,28,29,30]. Here, we characterized 283 *EjLRR-RLK* genes far more than those in *A. thaliana* and *O. sativa* [7]. The main reason may be that *E. japonica* possesses a large genome size of 749.3 Mb and 17 chromosomes, compared to *A. thaliana* with 125 Mb and 5 chromosomes as well as *O. sativa* with 430 Mb size and 12 chromosomes. The identified *EjLRR-RLK* genes carried both the LRR repeats and RLK domains although most of the subclasses had a Pkinase domain and LRRNT_2 (leucine-rich N-terminal). Prior research indicated that the Pkinase domain is evolutionarily conserved from *E. coli* to humans [37] and functions in diverse cellular processes, including divisions, cell proliferation, differentiation, and apoptosis [37]. The transcription of the RLKs has also been shown to be regulated in response to stress at different levels of gene responses [38]. To support this phenomenon, other RLKs have been shown in various cellular processes including the FSL2-, EFR-, and CERK1-mediated signaling pathways that modulate PTI activation triggering plant immunity [39]. This finding suggests that the *EjLRR-RLKs* may possess an extended range of functionality.

Variations in gene family sizes are often associated with gene duplication events [31,40]. In this research, we inferred that the size of the *EjLRR-RLK* gene family was, to a larger extent, affected by both the genome and duplication events [40]. According to earlier research, tandem and segmental duplications are two important types of gene duplication events that account for over 50% of all gene family expansions in the Rosaceae gene classes [28]. We observed that tandem and segmental duplications contributed 16% and 79%, respectively, toward the expansion of the *EjLRR-RLK* gene family. An in-depth investigation revealed that the tandem duplication was greater in specific classes and subclasses. Particularly, the classes (Class XI) with the highest number of *EjLRR-RLKs* were also the ones that had grown due to tandem duplications. Additionally, the collinearity analysis showed that *E. japonica* had more orthologous genes with *A. thaliana* as compared to *M. domestica* and *O. sativa*. This finding offers insight into the possible functions of the *EjLRR-RLKs* by relating to their characterized homologs in *A. thaliana.*

The Ka/Ks values demonstrated a Ka/Ks ratio less than 1, signifying a purifying selection except for *EjXIIIa-7* linked with *EjXIIIa-8*, which showed a positive selection, suggesting that these genes showing purifying selection might be part of the genes that contributed to the *E. japonica* adaption to external environmental stress [41]. 

The phylogenetic and evolutionary analysis of protein classes aids in the comprehension of the progressive development and function of the proteins [42]. In this research, the phylogenetic analysis of the LRR-RLK proteins in *E. Japonica* showed proteins clustering into 21 classes and subclasses (I–XV); however, some classes were absent. Additionally, this classification was based on similarities in the protein domain and configurations. The highest number of proteins was found in classes III and XI, indicating a rather high level of protein duplication in these classes. The fact that EjLRR-RLKs and other LRR-RLKs from different plant species, such as *A. thaliana*, clustered within the same classes, signifies that these proteins may have comparable functional roles. Additionally, EjLRR-RLKs were fully represented in almost all phylogenetic classes, implying a wide range of gene functionality. Interestingly, some EjLRR-RLKs clustered together with *P. patens*, suggesting that these genes are well conserved, since *P. patens* has become a plant model of choice, providing clues to plant mechanisms essential in the eco-evo-devo biology in plant fields [43,44]. 

A plant’s ability to acclimate to various developmental processes and environmental stimuli is influenced by its gene structure, which plays a significant role in the evolution of multigene classes [45]. The gene structure analysis of the *EjLRR-RLK* gene family members revealed differences that were consistent with the 15-family member classification (I–XV) and the phylogenetic relationships. Exon and intron numbers varied with gene sequences, as revealed by gene analysis in this study. Classes Xb-2, Xa-1, VII-2, XII, XI-1, III, IX, and Xb-2, for example, had exon ranges between 1-3 accompanied by 2 or 1 introns flanking the N- and C- terminals, whereas the remaining gene classes had more than 3 exons. The development of introns is an important process in genome evolution and an adaptive measure in species evolution [46]. The induction and quick processing of proteins following a stress response has been linked to an intronic deletion in the genes. Thus, this deletion has also been linked to the fast expression of genes under different stress responses. 

The LRR-RLKs are critical in numerous biological activities, including growth and development, and stress responses. Primarily, their interactions with other proteins and amongst themselves are vital for their function [47]. Thus, hetero- and/or homodimerization activates the LRR-RLKs [47]. For instance, heterodimerization between *BRASSINOSTREOID INSENSETIVE 1* (*LRR-RLK BAK1*) and its co-receptor *BRI1-ASSOCOATED RECEPTOR KINASE*; brassinosteroid signaling is essential in plant growth and development [48,49]. In this study, we showed that EjLRR-RLKs are densely interconnected, possibly to relay signals to the downstream components. In other studies, BAKI1 was also shown to interact with ER or ERL1 to regulate stomatal patterning [50]. 

In addition, plant *LRR-RLKs* constitute essential membrane-localized receptors that control plant development by sensing a variety of ligands. However, some RLKs occur in other places, such as the wall-associated kinases (WAKs) linked to the pectin fraction of the cell wall [51] and cytoplasmic-type RLKs localized in the cytoplasm [52]. In this study, we examined the cis-regulatory elements found in the identified *LRR-RLK* gene promoter regions. The biotic and abiotic stress response elements, as well as growth and development, were abundant in the promoter regions of the *EjLRR-RLKs.* A larger percentage was attributed to the stress-responsive classes, these included DRE, LTR, ARE, STRE, MYB, and MYC, suggesting that the *EjLRR-RLK* genes are actively involved in the transduction of environmental stress signals. DRE cis-elements have also been shown in *A. thaliana* to regulate cold- and drought stress similar to the C-repeat and low-temperature-responsive promoter elements [53]. 

To supplement the analysis, we performed the Gene Ontology (GO) analysis using data readily available on the Rosacea depository. Retrieved datasets were categorized into three terms: Biological Processes, Molecular Functions, and Cellular Processes. Most of the *EjLRR-RLK* genes were present in protein phosphorylation attesting to the previous fact that *EjLRR-RLK* genes are well conserved, and they carry the PKinase domain. Research has also shown that protein phosphorylation usually results in a functional change in the target protein by changing enzyme activity, cellular location, and protein-to-protein associations [54]. Other GO terms had a greater representation of *EjLRR-RLK* genes in ATP binding and protein binding processes, also suggesting that the *EjLRR-RLK* genes actively link with other proteins for effective gene function and that they are implicated in diverse cellular functions. In other studies, the RLKs have been revealed to converge with the receptor-like proteins (RLPs), triggering different responses and gene transductions [55].

Most important to this research is the gene expression analysis of the *EjLRR-RLK* genes. Research has shown that *LRR-RLK* genes are essential for plant adaption through regulating the abiotic stresses [24]. To confirm this assertion, we performed an RT-qPCR and analyzed the expression levels of 14 *EjLRR-RLKs*. Our results revealed that at least 50% of the analyzed *EjLRR-RLK* genes are upregulated during cold, drought, salt, and heat stress. Notably, *EjapXI-1.6* and EjapI*-2.5* were highly upregulated during the cold stress, while *EjapXI-1.10 and EjapIII-1* were highly upregulated in salt stress. Several RLKs in Arabidopsis from distinct subclasses are also reported to modulate salt stress, including *RPK1*, *CYSTEINE-RICH RLK (CRK36)*, *PROLINE-RICH-EXTENSIN-LIKE RLK4 (PREK4)*, and the *GUARD CELL HYDROGEN PEROXIDE-RESISTANT 1 (GHR1)* [38,56,57,58,59,60]. In *M. pohlia nutans*, *PnLRR-RLK* exhibited increased tolerances to salt stress and ABA stress, which doubled with an increase in *HKT1*, *SOS3*, *P5CS1*, and *DH1* salt tolerance genes. In addition, ABA stress was regulated through a decrease in expression of the ABA synthesis genes, like *NCED3*, *ABA1*, and *AAO3*, and ABA early response genes, *MYB2*, *RD22*, *RD29A*, and *DREB2A* [24,61], suggesting that *EjLRR-RLKs* may also regulate salt stress by interfering with the ABA-signaling pathway

Drought is a major abiotic stress affecting plant growth and distribution. Therefore, ABA acts as a key mediator of the drought stress response regulating the expression of osmotic response genes, physiological response, and plant growth. An LRR-RLK protein HSL3 negatively regulates stomatal closure by varying the amount of H_2_O_2_ in guard cells; as a result, it was determined that HSL3 is involved in the regulatory response to drought stress [19]. Rice’s *PHYTOSULFOKINE RECEPTOR* (*PSKR*) was elevated by ABA, which also improved Arabidopsis’ resistance to drought by increasing stomatal closure and controlling ROS activity in guard cells. Subsequent investigations revealed that *OsPSRR15* increases drought stress by directly interacting with *AtPYL9* and *OsPYL11*, its orthologue, through the nucleus and plasma membrane’s kinase domain [62,63]. Nonetheless, a few genes were upregulated in the drought and heat stresses here. Therefore, we concluded that different *EjLRR-RLK* genes regulate abiotic stresses differently. 

We also exhibited that the *EjLRR-RLK* genes respond to cold stress. Previous research has also demonstrated the regulation of cold stress by the *LRR-RLK* genes. For instance, In *G. soja*, *GsLRPK*, an *LRR-RLK* gene, was shown to interact with cold-responsive genes downstream to regulate and increased cold stress tolerances in Arabidopsis. Further investigation into the biochemical reactions of the *GsLRPK* showed kinase activities [64]. The *PHLOEM INTERCALATED WITH XYLEM-LIKE 1* (*PXL1*) was shown to be also essential in the regulation of the cold stress through the regulation of the activities of the ROS. In addition, *PXL1* phosphorylates the *HISTIDINE-RICH DEHYDRIN1* (*HIRD1*) and the *LIGHT-HARVESTING PROTEIN COMPLEX 1*, which further activates the downstream cold-responsive genes [65]. Research has also demonstrated that the *LRR-RLK* genes regulate different abiotic stresses by interacting with several pathways such as the MAPK cascade (through sequential phosphorylation reactions), the ABA-signaling pathways, and the Ca^2+^-influx, suggesting that the EjLRR-RLKs may also regulate abiotic stress by interacting with these pathways or more. In *M. truncatula*, a novel *LRR-RLK* gene *MtCTLK1* showed increased cold tolerance, which was accompanied by an increase in antioxidant enzymatic functions and proline accumulation; additional investigations also showed higher transcript levels of *CBFs* and CBF-dependent cold-responsive genes, showing that the *LRR-RLK* genes may regulate cold stress through the CBF pathway [65,66,67]. In Arabidopsis, the AtLRRop2 carrying four LRR motifs was shown to regulate cold stress due to the repeat LRR motifs and their protein-to-protein interactions [68]. A wheat *LRR-RLK* gene, *SOMATIC EMBRYOGENESIS RECEPTOR KINASE* (*SERK*), interacts with *TaDJA7* to activate the *HTSPs* and has a dual role in heat tolerance and biotic stress resistance against *P. striiforms F.* sp. *Triitcii* [23]. Another *LRR-RLK* gene *PHLOEM INTERCALATED WITH XYLEM-LIKE 1* (*PXL1*) in Arabidopsis is responsive to heat and cold stress and phosphorylates *AtHIRD1* and *AtLHCA1* to regulate the signal transduction pathways in response to temperature changes [65]. 

Wholly, this research can be summarized; thus, *EjLRR-RLK* genes are a large gene family that has expanded probably due to gene duplications. Through adaptions, they have diversified into several classes that perform different roles including regulation of the abiotic stresses. 

## 4. Materials and Methods

### 4.1. Identification and Classification of EjLRR-RLK Gene

To identify the *LRR-RLK* gene family in the *Eriobotrya japonica*, the *E. japonica* genome and genome resources were mined from the *Eriobotrya japonica* v1.0 GDR (https://www.rosaceae.org/Analysis/14720732; accessed on 21 March 2023) [69]. The *A. thaliana LRR-RLK* genes obtained from TAIR (https://www.arabidopsis.org/browse/genefamily/leuc.jsp; accessed on 21 March 2023) served as a reference. Using the PBLAST tool available on the website and a standard HMMER search in Tbtools [70] with an E-value cut-off of 0.0001, putative protein kinases (PKs) were discovered. The Hidden Markov Models of the typical Pkinase clade [Pkinase (PF00069) and Pkinase_Tyr (PF07714)] were obtained from the Pfam database v.28 [71]. Typical protein kinases were considered to have at least 50% coverage of the Pfam domain model [28]. Following this screening, putative EjLRR-RLKs were further authenticated using SMART (http://smart.embl-heidelberg.de/; accessed on 21 March 2023) and CDD search from the NCBI CDD (Conserved Domains Database (CDD) and Resources (nih.gov)). Furthermore, the detected protein kinases (PKs) were categorized into classes and subclasses at an E-value cut-off of 0.0001 using previously described HMMs of various typical PK classes and subclasses [72]. The phylogenetic classification of model plant species *A. thaliana*, *O. sativa*, *P. patens*, and *C. reinhardtii* was used to authenticate this HMM subfamily classification [72]. *LRR-RLK* genes of other plant genomes including *Marchantia polymorpha*, *Oryza sativa*, and *Vitis vinifera* were obtained from the Phytozome v13 (https://phytozome-next.jgi.doe.gov/, accessed on 21 March 2023) and previous publications (Supplementary File S5).

Furthermore, the DeepLoc-2.0 program (https://services.healthtech.dtu.dk/services/DeepLoc-2.0/; accessed on 28 April 2023) was used to identify the subcellular localization of all identified LRR-RLK proteins. Previous techniques employed by Hwarari et al. [31] were used to examine the conserved domain (CDD), motif number and configurations, and cis-elements. 

### 4.2. Multiple Alignments and Phylogenetic Analysis of EjLRR-RLK Genes

The phylogenetic classification of protein sequences provides essential information related to protein evolution and possibly function. In this study, we used full-length LRR-RLK protein sequences from four plant species, *Marchantia polymorpha*, *Oryza sativa*, *Vitis vinifera*, and *Eriobotrya japonica*, to construct a phylogenetic tree. The MUSCLE program in Mega X was used to perform multiple alignments and a maximum likelihood phylogenetic tree was built at a 1000 bootstrap value. To validate the ML phylogenetic tree, the neighbor-joining tree (NJT) approach was used to construct a phylogenetic tree using MEGA X software based on amino acid alignments. The p-distance model was used with a replication bootstrap value of 1000 times. Then, the Evolview [73] and iTOL online tools (iTOL: Interactive Tree Of Life (embl.de)) were employed to enhance the appearance of the tree [74].

### 4.3. Chromosome Location, Gene Duplication, and Synteny Analysis of EjLRR-RLK Genes

The gene location information was retrieved from the genomic resources and further mapped using the Tbtools program. The gene synteny analysis is crucial in understanding gene evolutionary relationships providing precise predictions on genome evolution and gene regulation and function. We used the default parameters of McScanX in Tbtools for synteny analysis and the synonymous and non-synonymous ratios were calculated using the Tbtools’ Ka/Ks calculator. By comparing the LRR-RLK gene in each chromosome’s or contig’s matching position, the tandem and segmental repeated genes were found, and neighboring genes were identified as tandem duplicated genes.

### 4.4. Cis-Regulatory Elements and Gene Ontology Analyses of EjLRR-RLK Genes

To analyze the abundances of cis-regulatory elements present in the E. japonica LRR-RLK genes, a 2kB expanse was regarded as the promoter region, and all the identified EjLRR-RLK genes were submitted to the PlantCARE database for additional predictions (http://bioinformatics.psb.ugent.be/webtools/plantcare/html; accessed on 21 March 2023). The results were visualized using a heatmap generated with the TBTools software. The gene ontology (GO) analysis provides a basis and set of models for analyzing and predicting gene functions, specifically designed for computational presentations [75]. To gain insight into the possible functions of EjLRR-RLKs, GO datasets of *E. japonica* were retrieved from the Eriobotrya japonica Genome v1.0 Assembly and Annotation database (https://www.rosaceae.org/Analysis/14720732; accessed on 14 June 2023). 

### 4.5. Protein-to-Protein Interaction and Three-Dimensional (3D) Protein Structures of EjLRR-RLK Proteins

The three-dimensional structure helps to predict the possible protein functions; by using the SWISS-MODEL database (www.swissmodel.expasy.org/; accessed on 27 April 2023), PDB files corresponding to the EjLRR-RLK protein were obtained using the homology modeling method. The PDB files were visualized using the Chimera X-1.61 software (http://www.cgl.ucsf.edu/chimera/; accessed on 21 March 2023). The obtained full protein sequences of *E. japonica* LRR-RLKs were further submitted into the STRING database (http://string-db.org, retrieved on 27 April 2023) to predict the protein interactions. The network was then beautified using the Cytoscape software (v3.9.1).

### 4.6. Plant Material, Treatment, and RT-qPCR Expression Analysis

Plant materials were collected from the Nanjing Forestry, College of Biology and the Environment, and grown under natural conditions in the loquat germplasm resource preservation garden. The seedlings used in this experiment grew to almost 0.6 m with at least 5 mature leaves and were divided into 4 batches of 3 seedlings per batch and 3 biological replicates. The seedlings were set to stress treatment. The batches were subjected to four different treatments: 4 °C for cold stress treatment, 40 °C heat stress, drought conditions set at 40% polyethylene glycol/PEG600, and salt stress saline solutions of 150 mM NaCl (Sodium Chloride) were added to the soil medium. Both tender and mature leaves were collected from the seedlings after 3 days of stress and then placed in liquid nitrogen (N) for quick freeze and stored at −80 °C for additional analyses. 

The expression patterns of EjLRR-RLK genes were analyzed under four abiotic stresses (cold, heat, drought, and salt) as treatments of both the treated and control plants using the RT-qPCR analysis. Total RNA extraction was performed on the *E. japonica* leaves using the KK-rapid plant total RNA extraction kit (Beijing Zoman Biotechnology Co., Ltd., Beijing, China). The quality of total extracted RNA samples was analyzed using agarose gel electrophoresis (Supplementary Figure S4) and RNA integrity number (RIN) was evaluated by Agilent 2100. The first-strand cDNA was generated using the acquired RNA and an Evo M-MLV RT kit (GDNA. CLEAN for QPCRII AG 11,711; Accurate Biotechnology Co., Ltd., Changsha city, Hunan province, China). Three replicates were used for the technical experiments. In addition, the Roche LightCycler^®^480 real-time PCR equipment (Solna, Sweden) SYBR-green was used for the qPCR investigations using the 2^−ΔΔCT^ method to determine the genes’ relative expression levels. Furthermore, the EF1α and UBCE genes were utilized as internal reference genes, and gene primers were designed using Primer 5.0 (Supplementary File S6). The melting curve graphs were used to ensure the accuracy and reliability of the qPCR results (Supplementary Figure S5). Additionally, all of the primers were examined using agarose gel electrophoresis, and their primer specificity was confirmed by PCR before RT-qPCR analysis. The RT-qPCR result was shown in GraphPad Prism-10. 

### 4.7. Statistical Approaches

The relative expression of each EjLRR-RLK gene is represented by the vertical bars; error bars were generated as mean ± the Standard Deviation (SD) of the three independent replicates. The statistical significance of the differences was validated by Dunnett’s multiple comparison test (* *p* < 0.05, ** *p* < 0.01, *** *p* < 0.001, **** *p* < 0.0001).

## 5. Conclusions

Plants continue to evolve to adapt to external abiotic stresses. The knowledge of plant gene structures and possible functions is crucial in aiding plants’ adaptation. In this study, we identified *283 LRR-RLK* genes in *E. japonica*, which we concluded to have increased due to two gene duplication events, segmental and tandem duplications. Additional investigation through gene structure, comparative phylogeny, and cis-acting elements exhibited a clustering into subclasses based on protein domain arrangements, intron, and exon numbers and arrangements. Additionally, we concluded that these attributes provide the basis of gene function and responses to abiotic stress. To ascertain this claim, we performed qPCR analysis of 14 *EjLRR-RLK* genes, which were selected based on cis-regulatory analysis. Interestingly, each family showed differing responses to the cold, heat, drought, and salt stresses. However, from this analysis, we concluded that *EjLRR-RLK* genes regulate abiotic stress. Wholly, this study can be summed as that: *LRR-RLK* genes are present and well conserved in the *E. japonica*. Additionally, they function to modulate the abiotic stresses at varying degrees; this attribute can be related to their gene structure arrangements and/or protein domain presence and arrangements.

## Data Availability

The genome and gene information used in this research are readily available at the *Eriobotrya japonica* database v1.0 GDR (https://www.rosaceae.org/Analysis/14720732; accessed on 21 March 2023). Those of *Arabidopsis thaliana* are readily available at (https://www.arabidopsis.org/browse/genefamily/leuc.jsp; accessed on 21 March 2023).

## Supplementary Materials

The following supporting information can be downloaded at https://www.mdpi.com/article/10.3390/plants13172387/s1. Supplementary Figure S1. Conserved analysis in EjLRR-RLK full protein sequences. Far left, the EjLRR-RLK NJT phylogenetic tree generated using the TBtools v 1.611. Followed by the conserved motif analysis of EjLRR-RLKs, motifs are numbered. Second from far right is the conserved domain analysis. Far right, shows the conserved exon-intron arrangements in *EjLRR-RLK* genes, defined in the top right corner. Supplementary Figure S2. Putative cis-elements present in the *EjLRR-RLK* genes showing their respective positions. Supplementary Figure S3. 15 Motif logos of EjLRR-RLKs searched through the MEME tool; accessed on 21 March 2023. Supplementary Figure S4. RNA quality investigation. M stands for Marker DL2000; 1–15: RNA product (1–3: CK; 4–6: Cold; 7–9: Drought; 10–12: Salt; 13–15: Heat). Supplementary Figure S5. qPCR melting curves of 15 representative EjLRR-RLKs illustrating the specificity of the amplified product. The single, shar peak indicates a specific product, while the absence of additional peaks confirms the lack of primer-dimers or non-specific amplification. Supplementary File S1. *Eriobotrya japonica* genome resource for known PKs and identified 1829 typical PKs. Supplementary File S2. The physiochemical properties of identified 283 EjLRR-RLK protein sequences, showing their gene, protein, and accession IDs. Supplementary File S3. The synonymous, nonsynonymous values, and their ratios of linked *EjLRR-RLK* genes. Calculated using the TBtools software v 1.611. Supplementary File S4. Putative cis-elements present in the *EjLRR-RLK* genes showing their respective positions. Supplementary File S5. LRR-RLK genes from other plants including *Marchantia polymorpha*, *Oryza sativa*, and *Vitis vinifera* obtained from the Phytozome v13 (https://phytozome-next.jgi.doe.gov/; accessed on 21 March 2023) and previous publications. Supplementary File S6. qPCR primers for *EjLRR-RLK* genes and the EF1-alpha.
