# Supplementary material for: Genomic Survey of LRR-RLK Genes in Eriobotrya japonica and Their Expression Patterns Responding to Environmental Stresses
Source: Plants (Basel). 2024 Aug 27;13(17):2387. doi: 10.3390/plants13172387 (PMC11397332; doi:10.3390/plants13172387)
Supplement: Supplementary file 1 [file plants-13-02387-s001.zip › Supplementary Figures/Supplementary Figure S4.pdf]

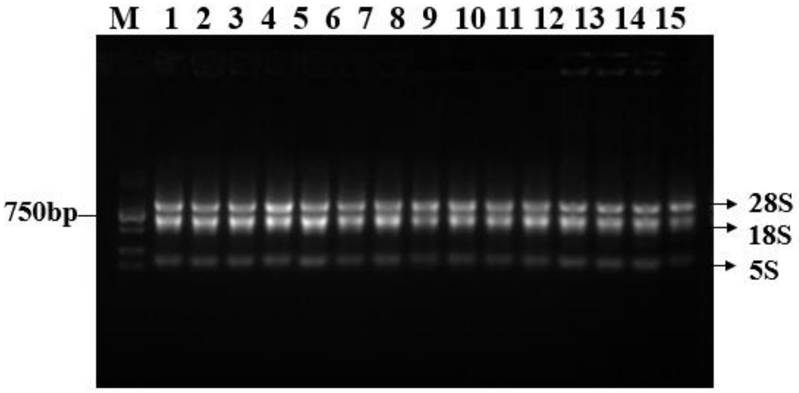

Figure S4. RNA detection of representative plant samples by agarose gel electrophoresis. M: Marker DL2000; Lanes 1-15: RNA product for each sample (1-3: CK; 4-6: Cold; 7-9: Drought; 10-12: Salt; 13-15: Heat).
