# Supplementary material for: Genomic Survey of LRR-RLK Genes in Eriobotrya japonica and Their Expression Patterns Responding to Environmental Stresses
Source: Plants (Basel). 2024 Aug 27;13(17):2387. doi: 10.3390/plants13172387 (PMC11397332; doi:10.3390/plants13172387)
Supplement: Supplementary file 1 [file plants-13-02387-s001.zip › Supplementary Figures/Supplementary Figure S5.pdf]

（a） *EjapI-11*

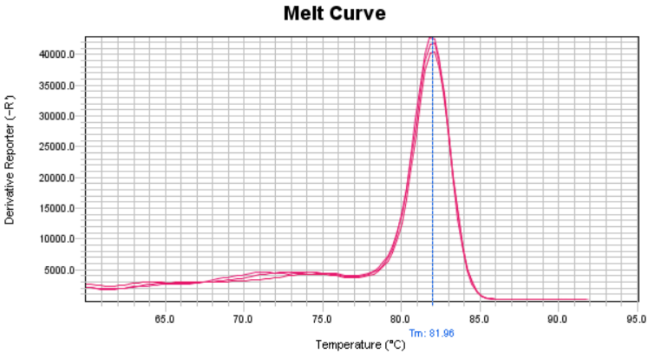

CK

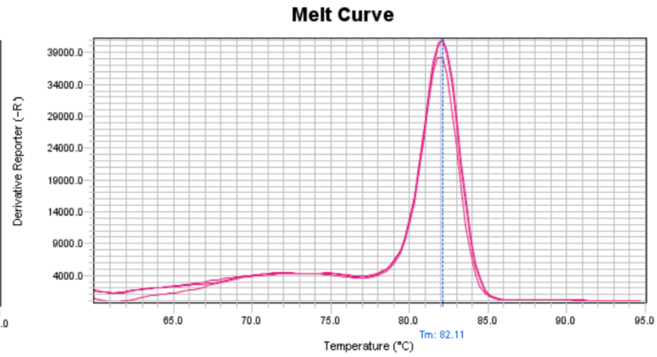

Cold

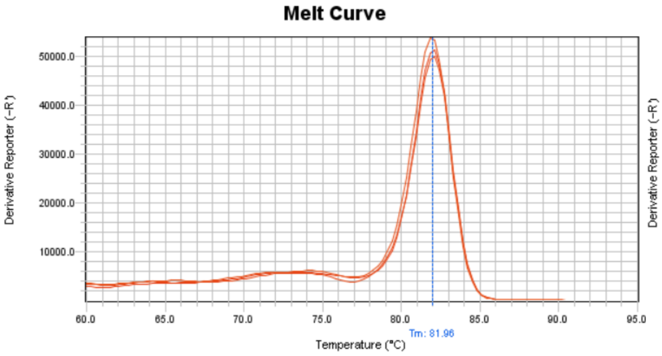

Drought

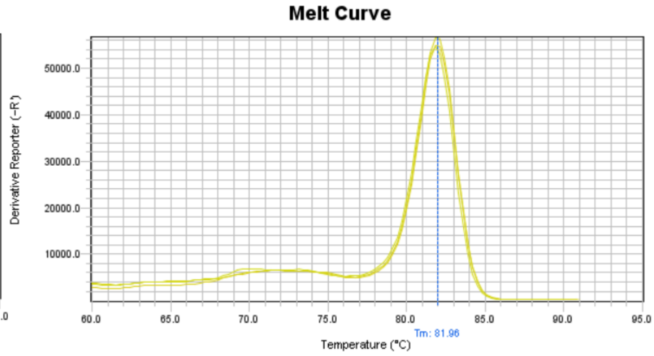

Nacl

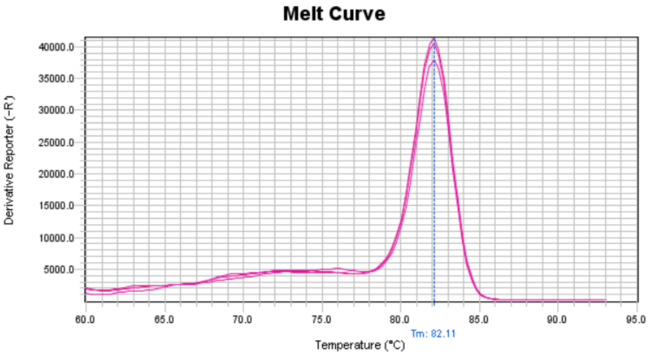

Heat

(b) *EjapXb-1.15*

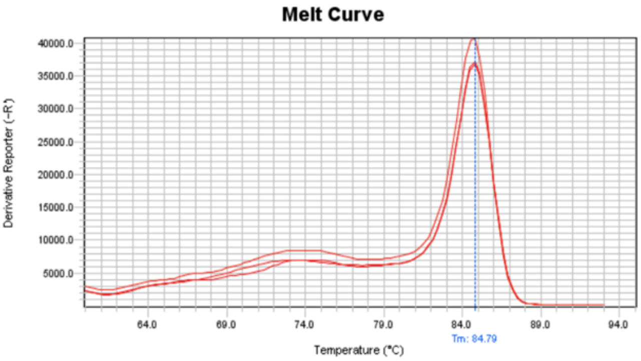

CK

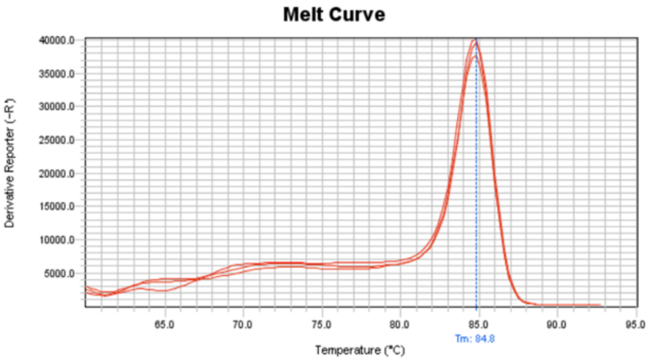

Cold

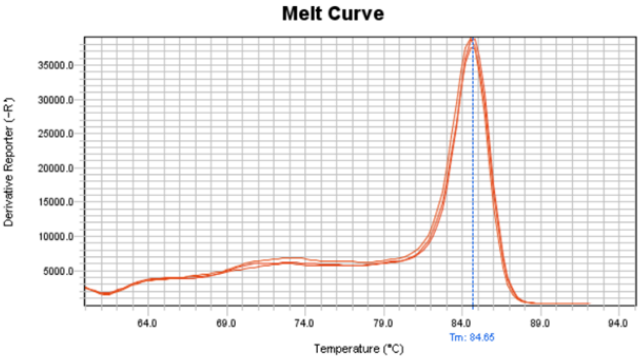

Drought

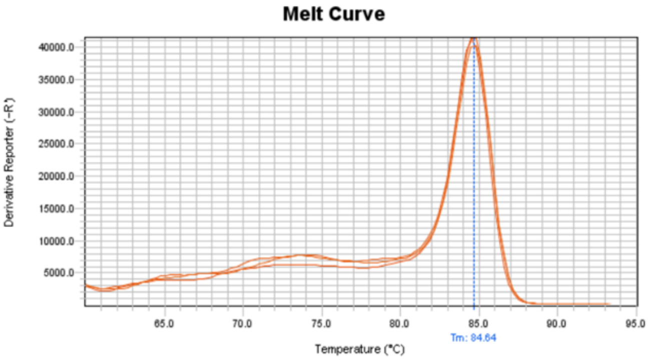

Nacl

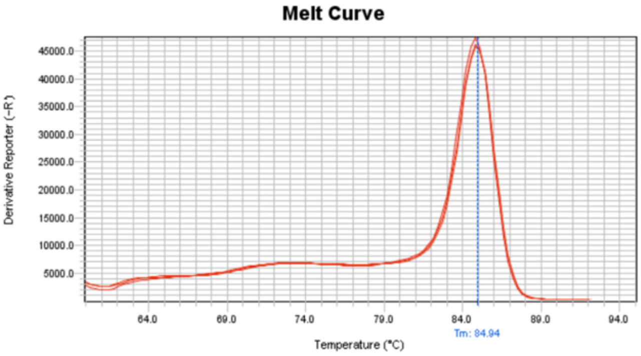

Heat

（c）*EjapXI-1.10*

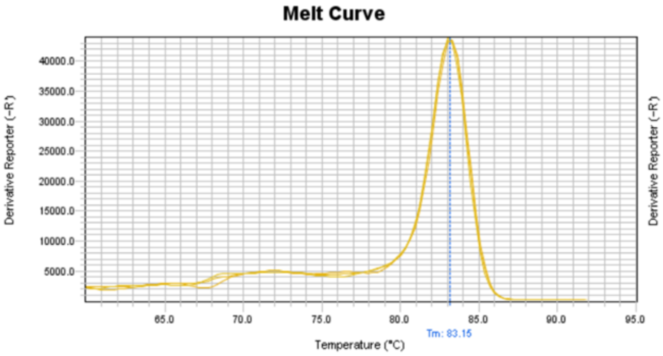

CK

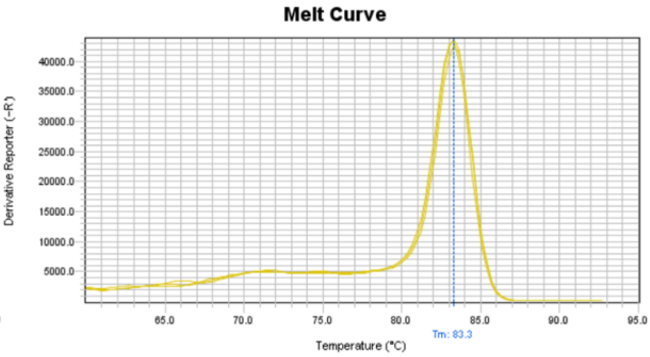

Cold

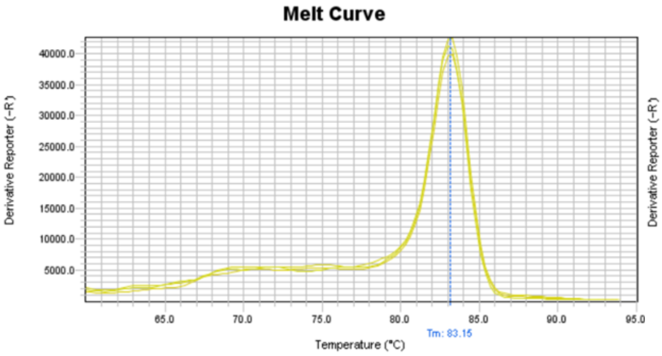

Drought

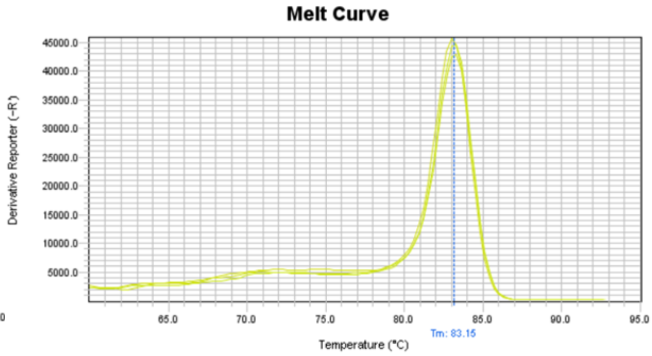

Nacl

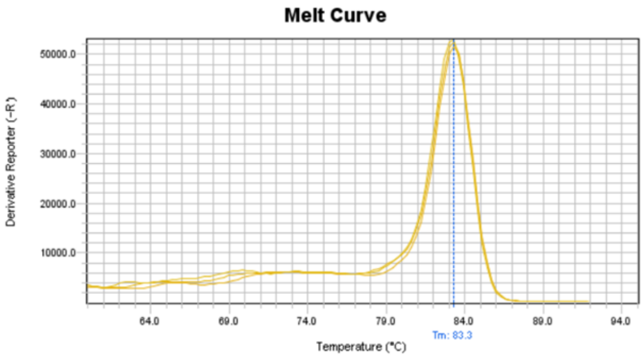

Heat

(d) *EjapXI-1.6*

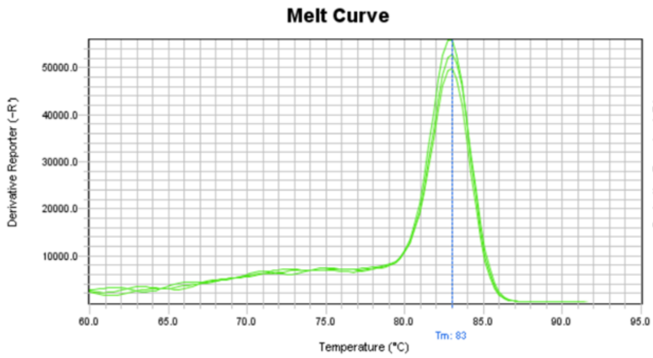

CK

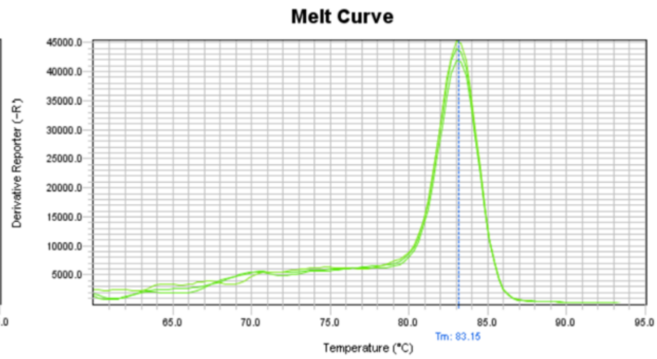

Cold

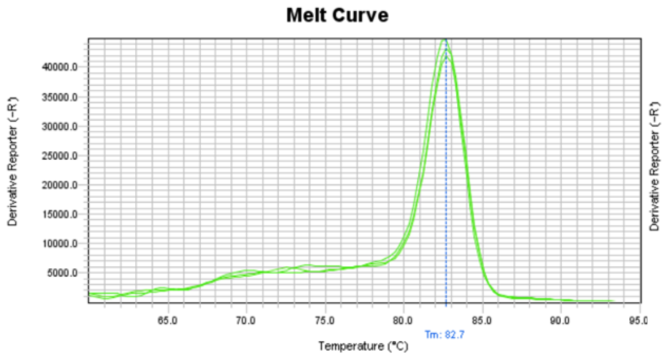

Drought

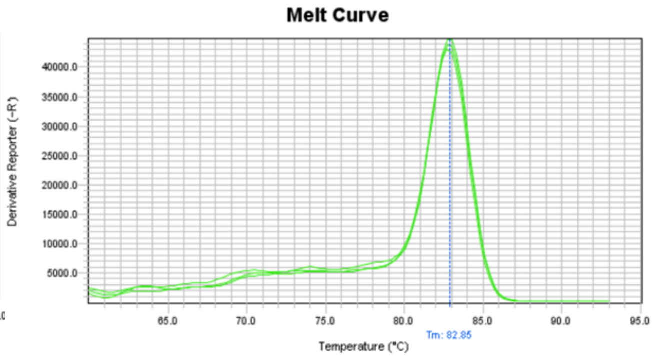

Nacl

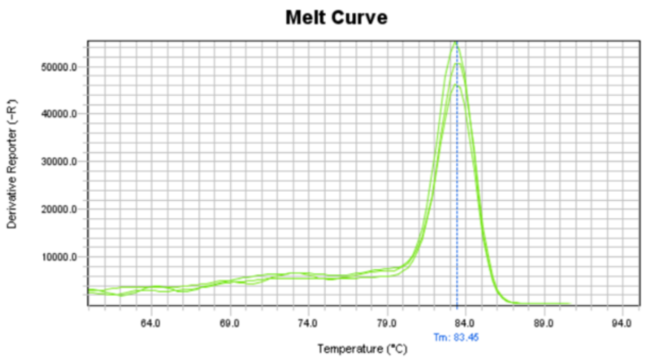

Heat

（e） *EjapXI-1.26*

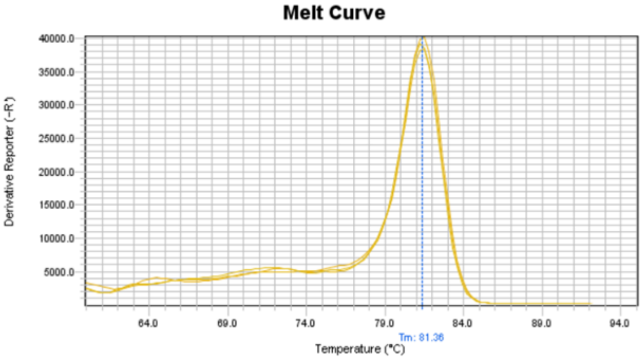

CK

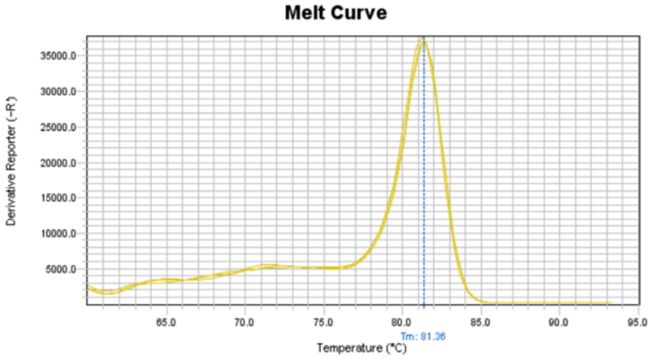

Cold

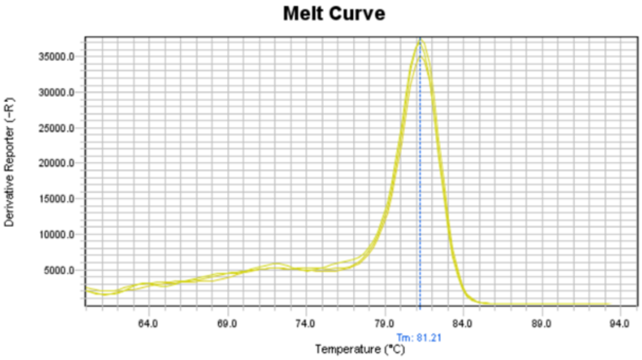

Drought

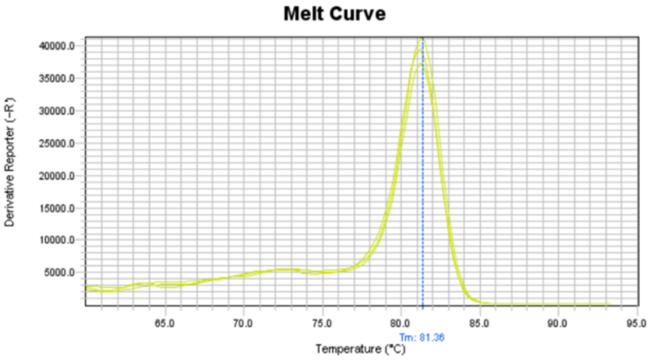

Nacl

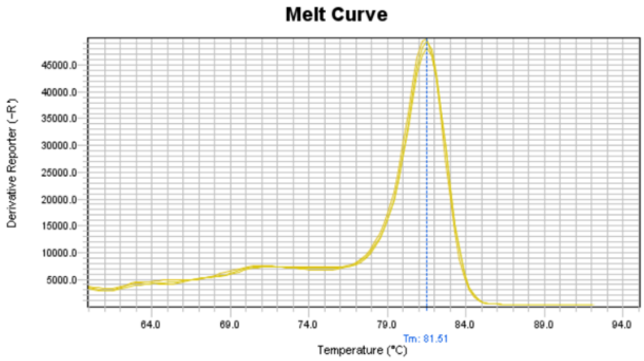

Heat

(f) *EjapV-2*

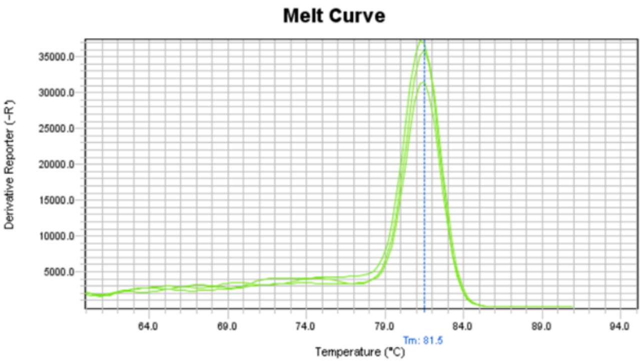

CK

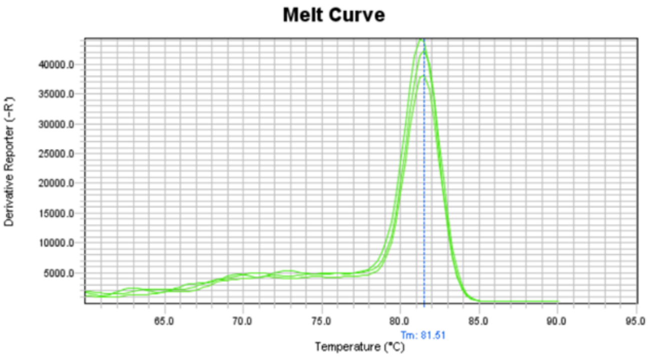

Cold

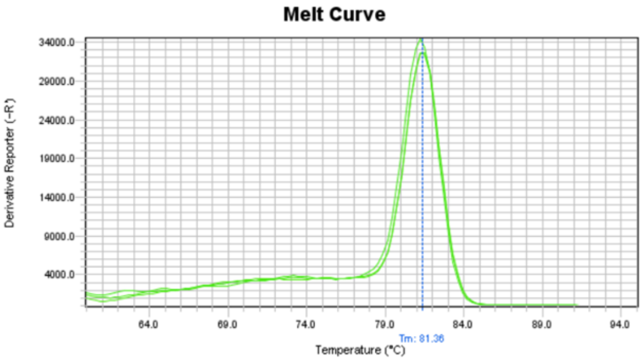

Drought

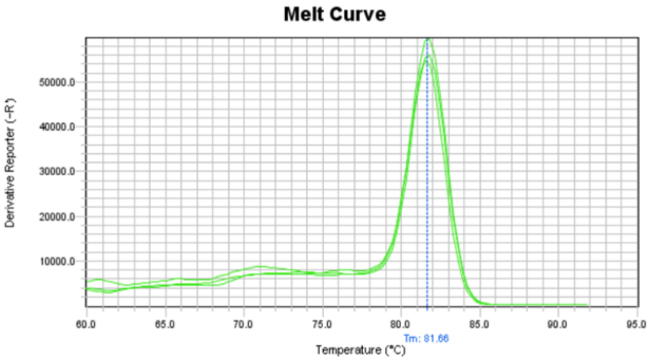

Nacl

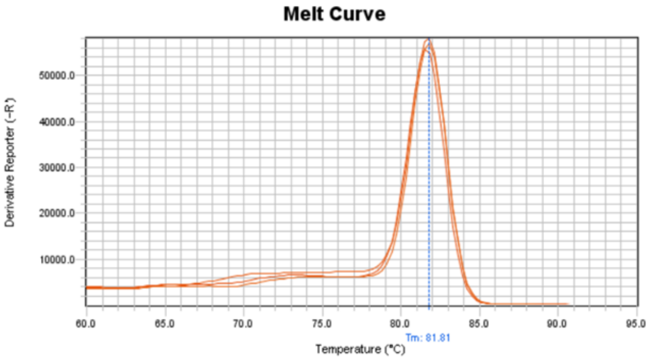

Heat

（g）*EjapIII-24*

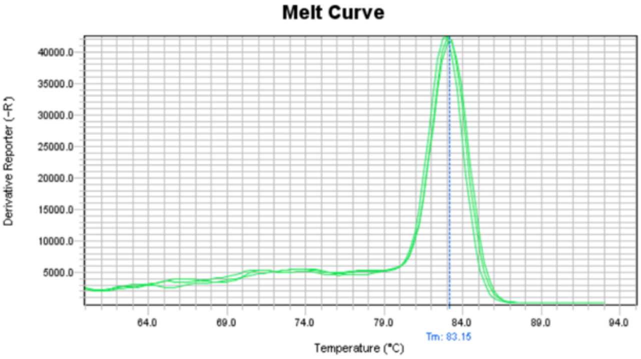

CK

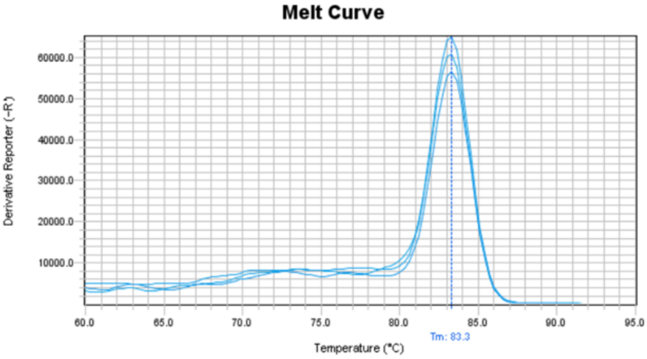

Cold

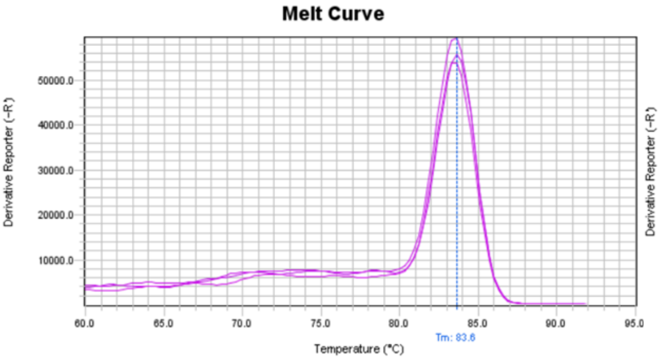

Drought

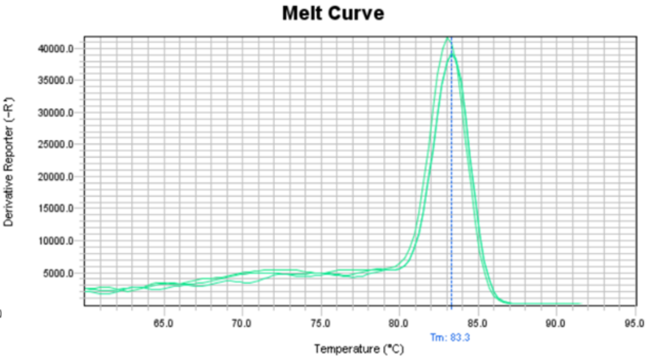

Nacl

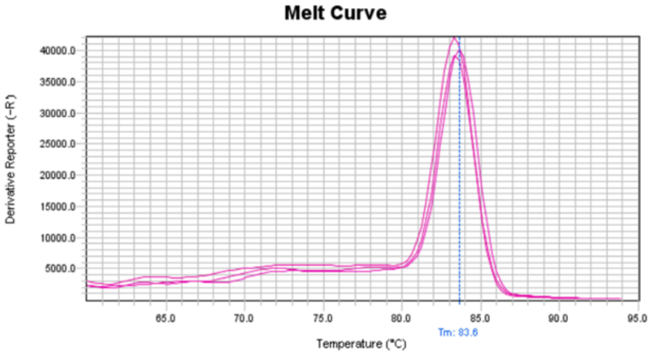

Heat

（h）*EjapII-2*

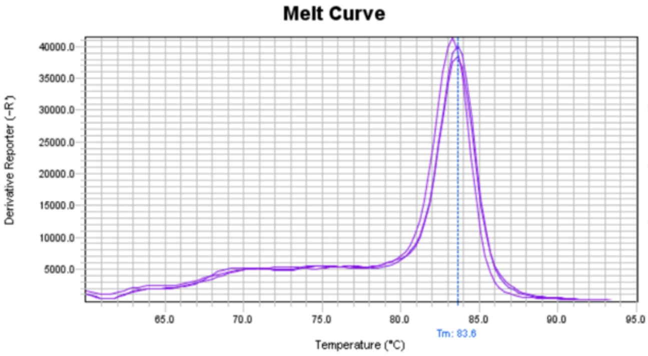

CK

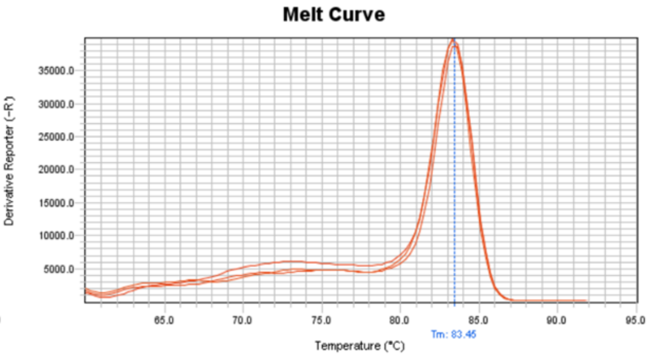

Cold

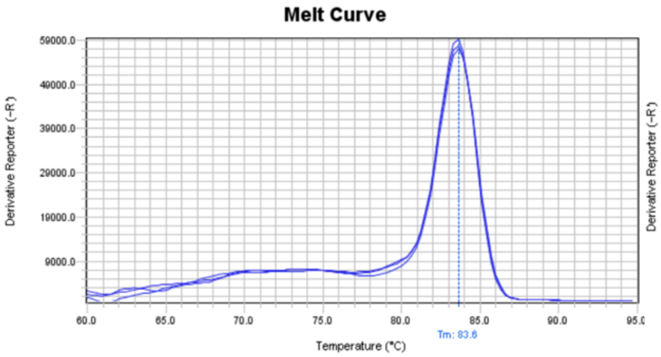

Drought

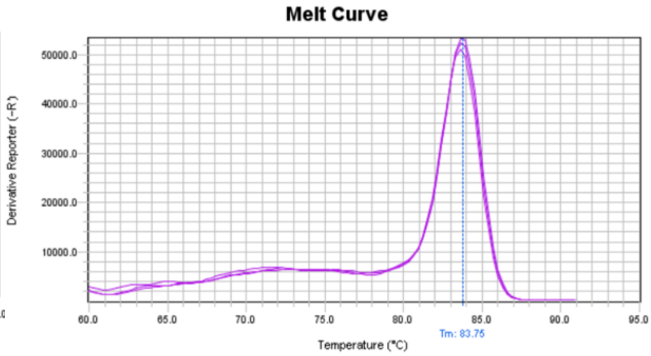

Nacl

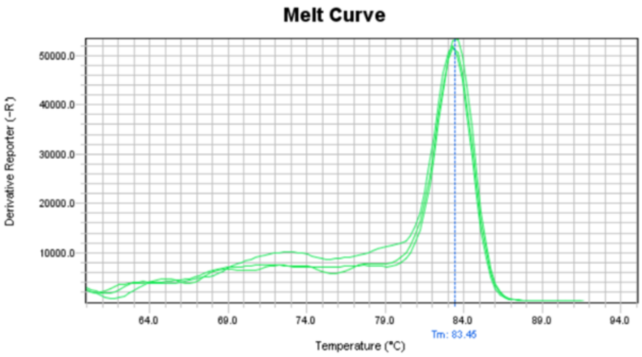

Heat

(i) *EjapI-2.5*

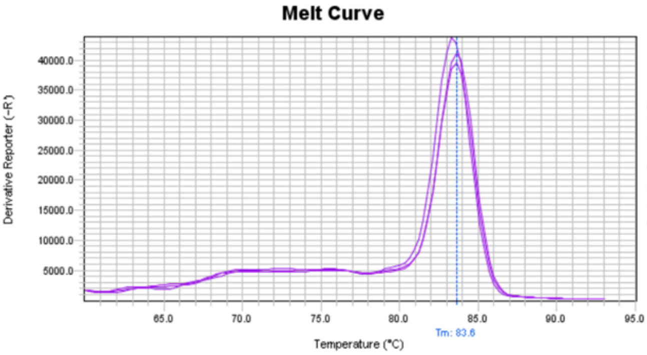

CK

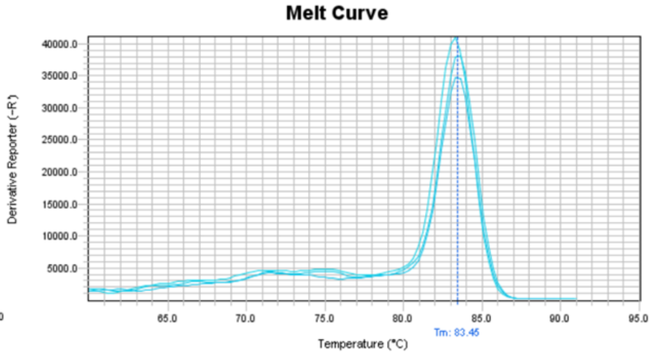

Cold

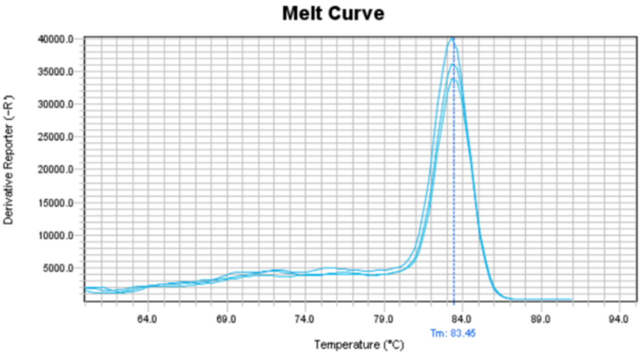

Drought

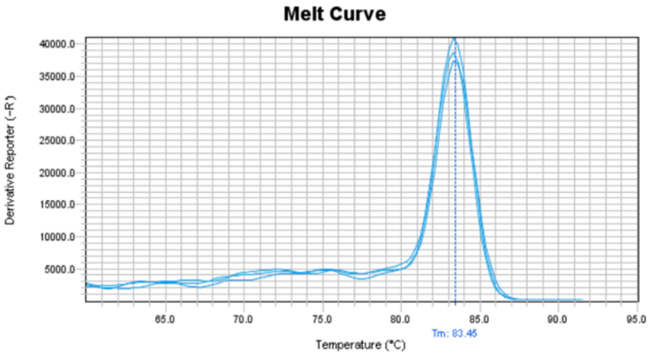

Nacl

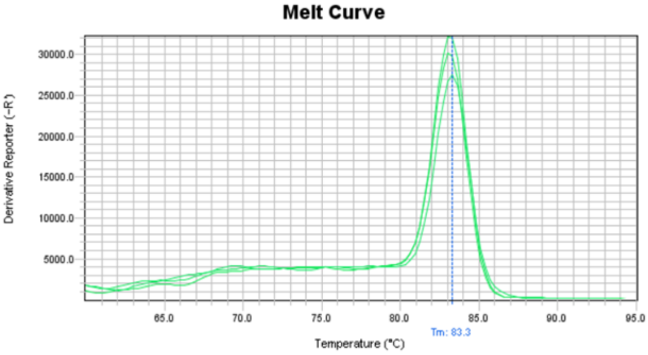

Heat

(j) *EjapXb-1.20*

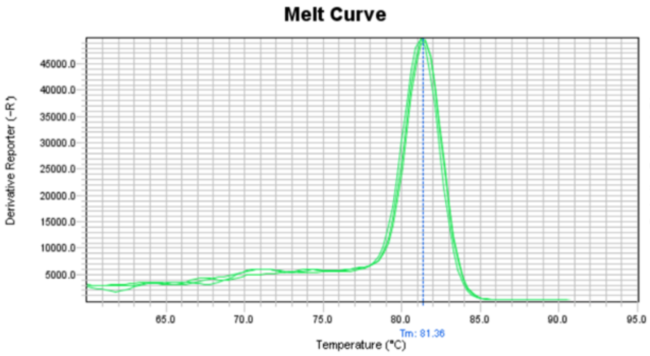

CK

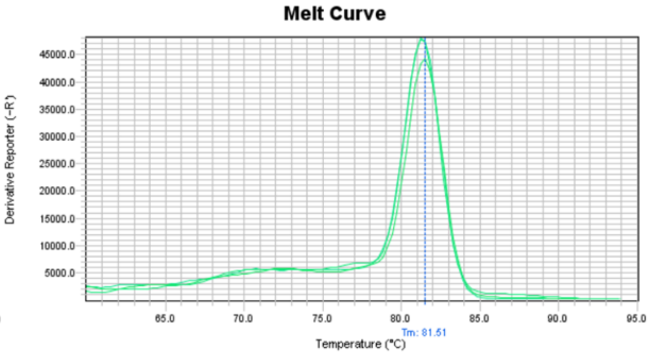

Cold

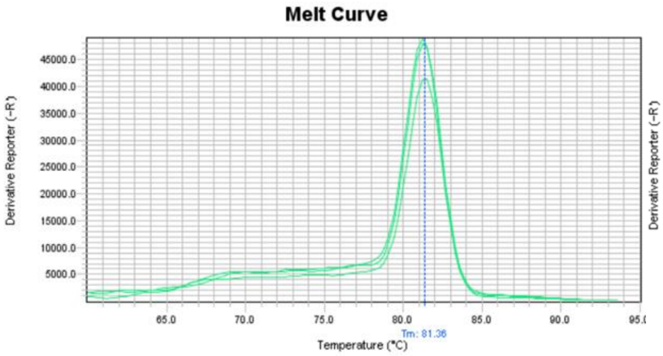

Drought

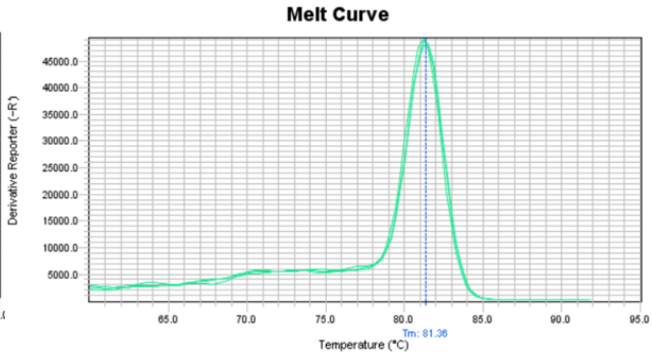

Nacl

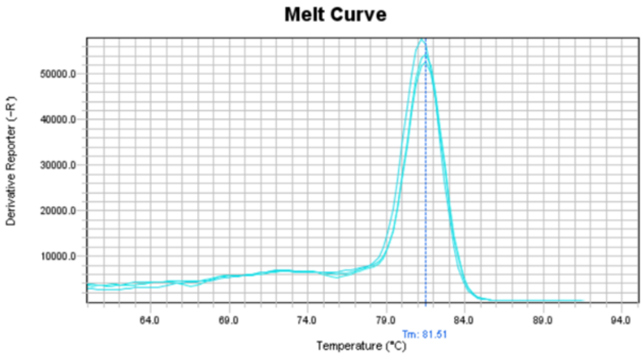

Heat

(k) *EjapIX-3*

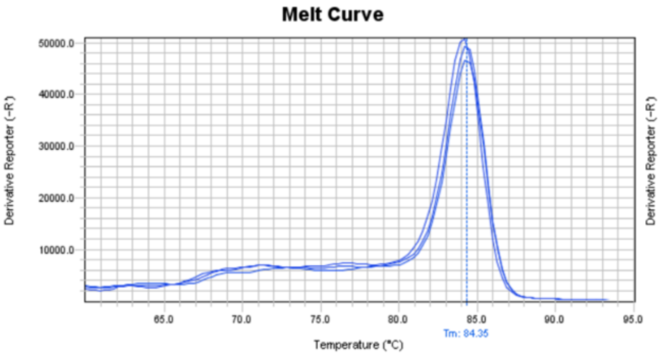

CK

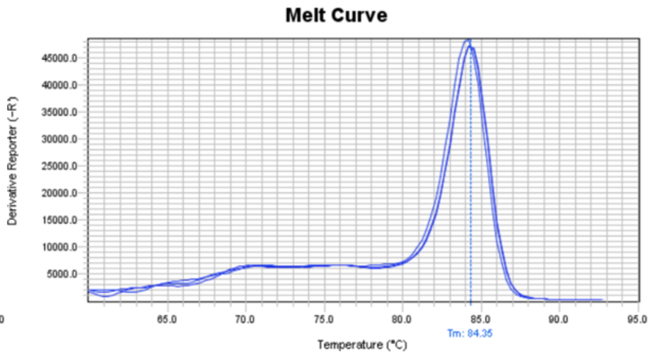

Cold

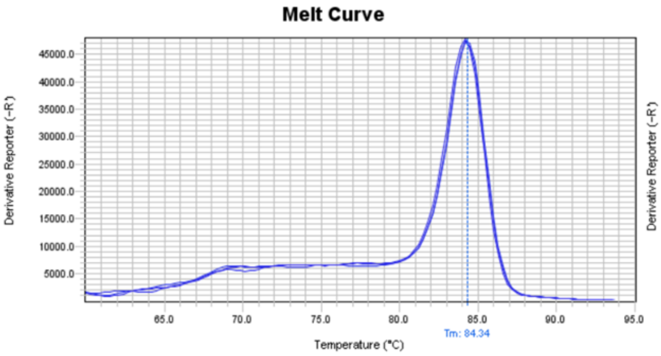

Drought

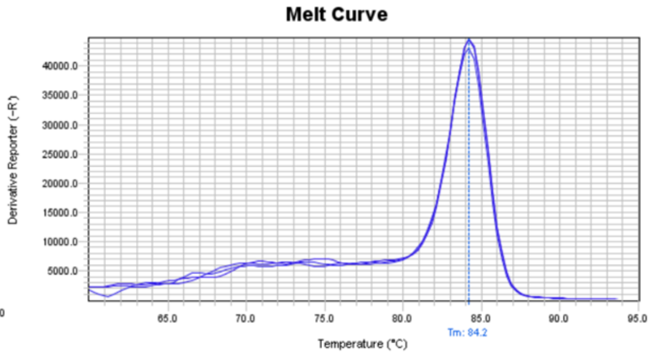

Nacl

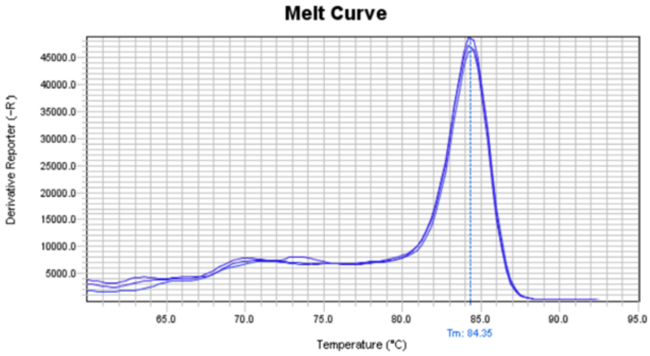

Heat

(1) *EjapIII-1*

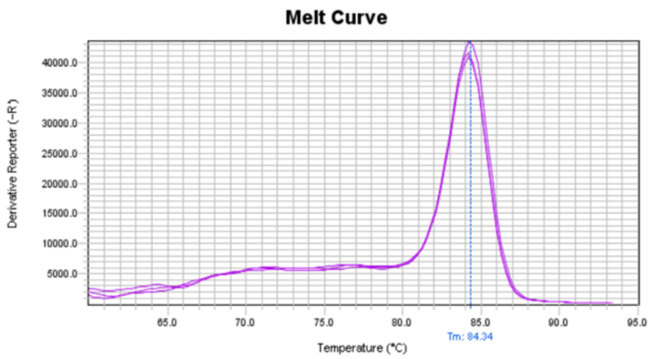

CK

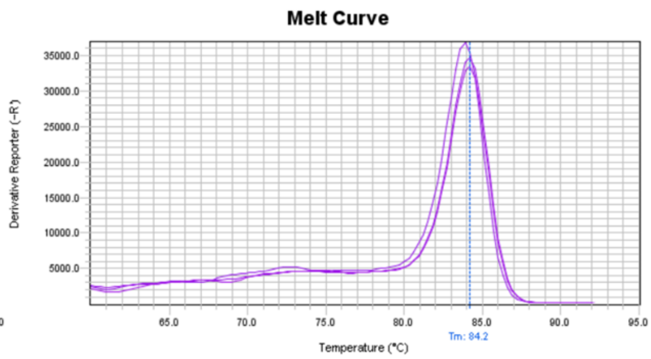

Cold

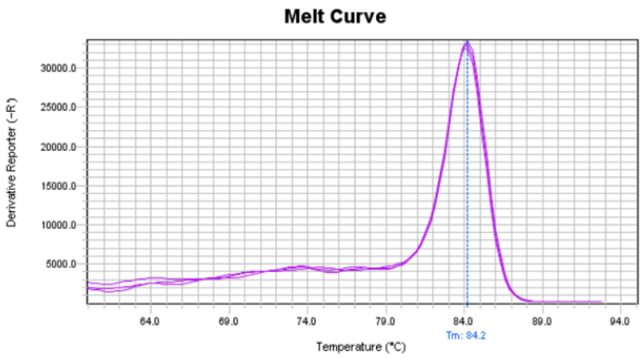

Drought

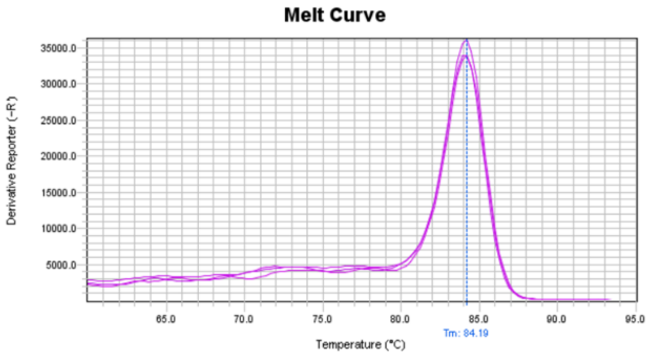

Nacl

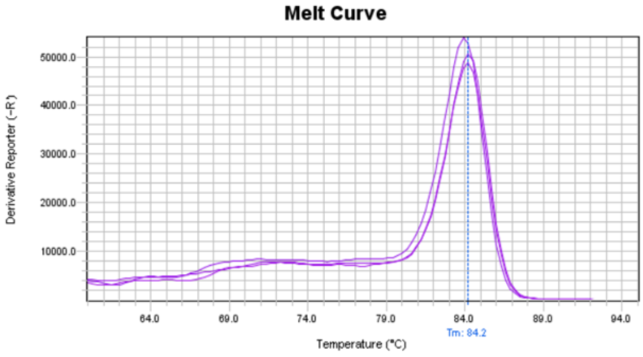

Heat

（m）*EjapVI-1.8*

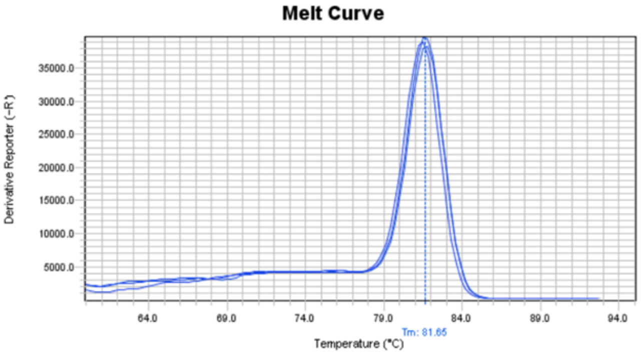

CK

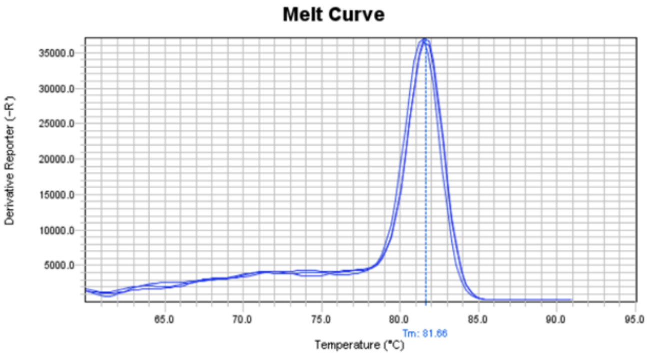

Cold

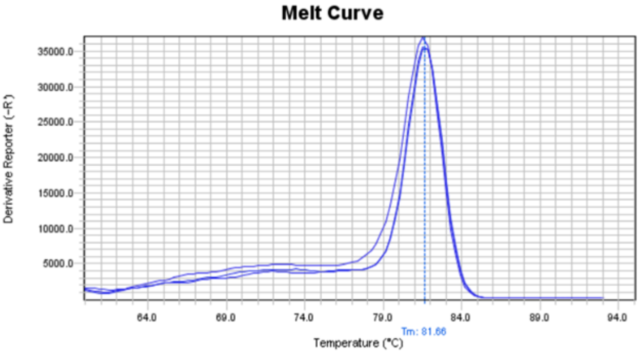

Drought

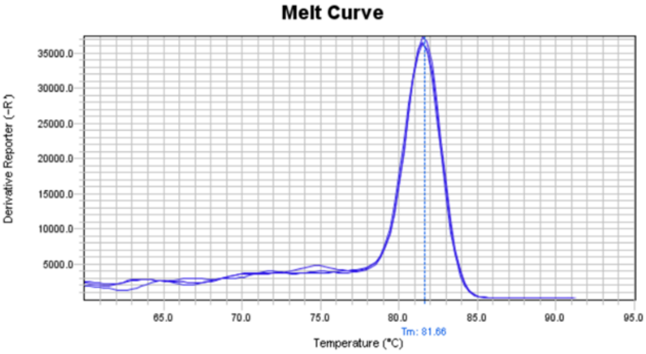

Nacl

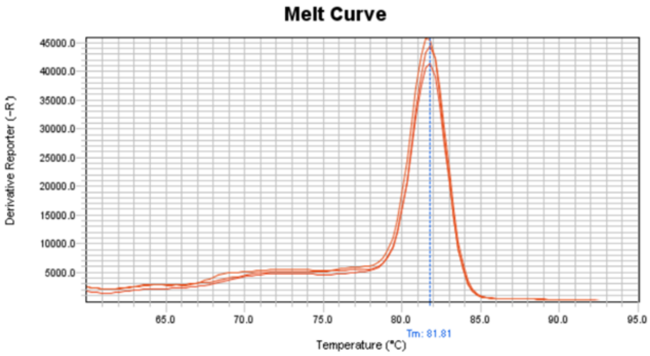

Heat

(n) *EjapV-10*

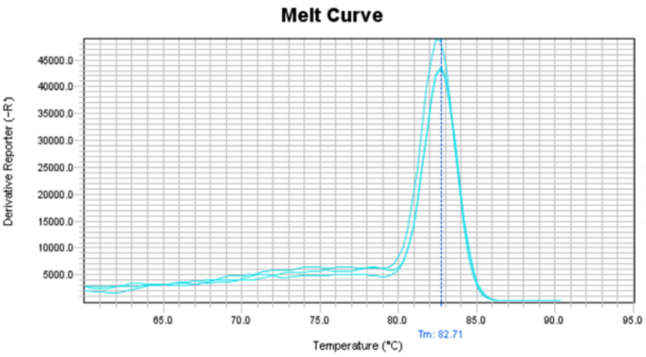

CK

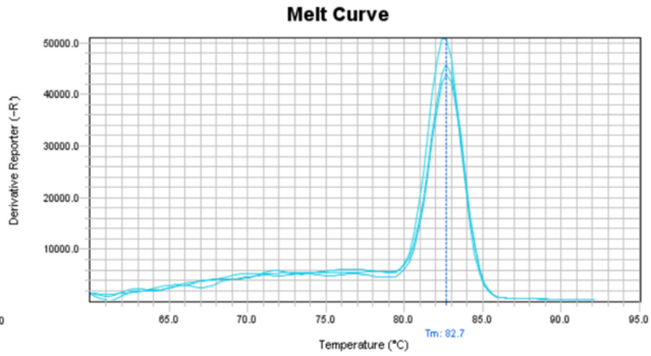

Cold

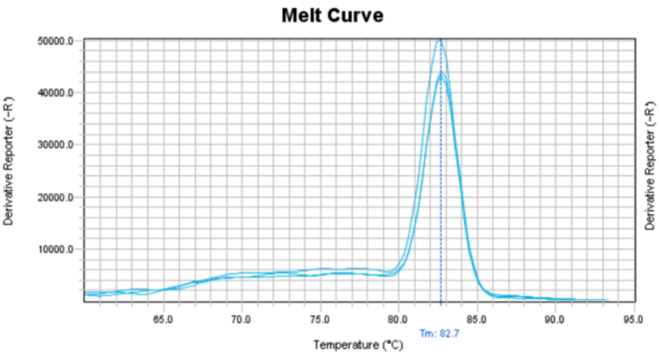

Drought

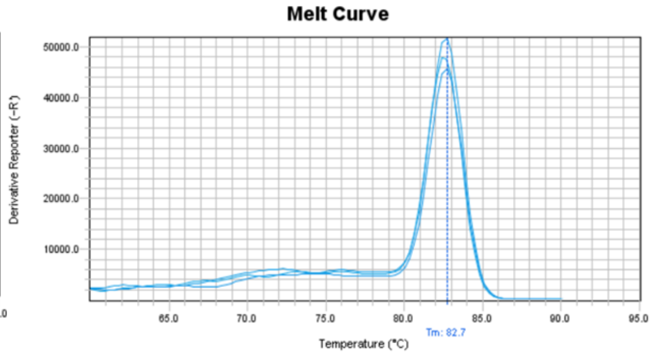

Nacl

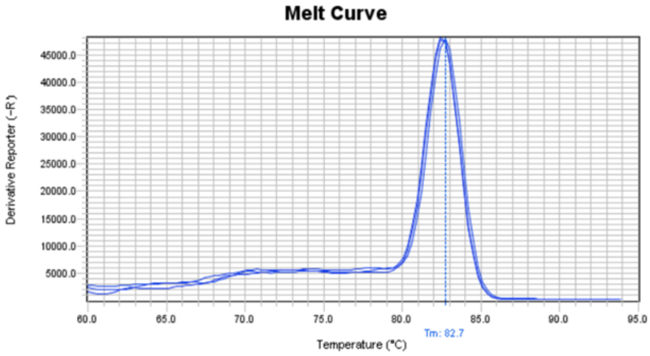

Heat

(o) *EF1-alpha*

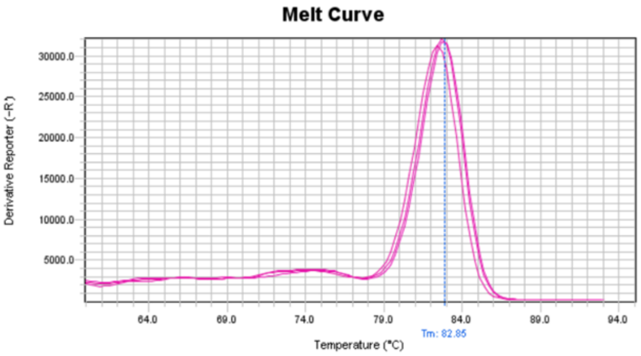

CK

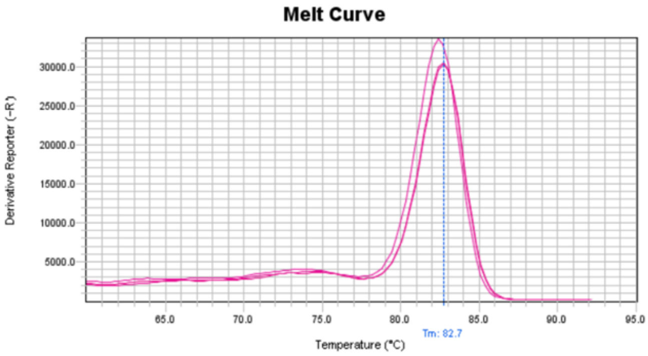

Cold

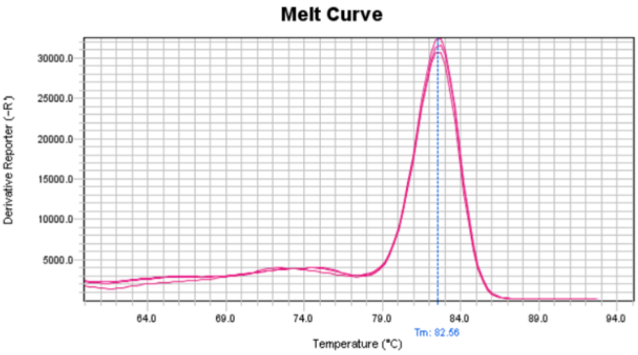

Drought

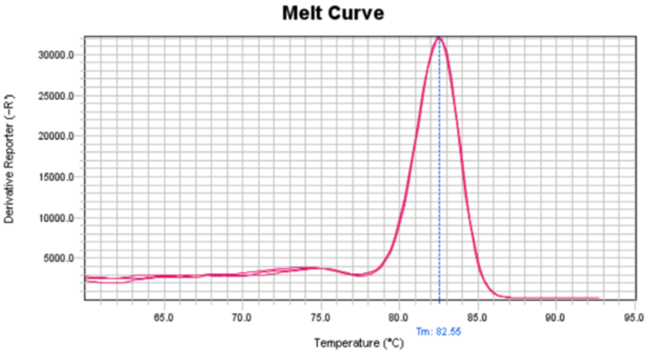

Nacl

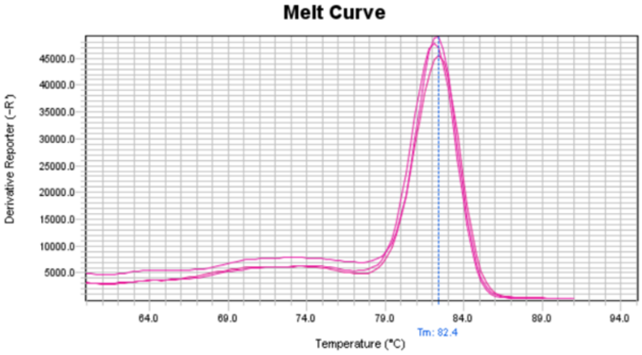

Heat

Figure S5. Melting Curve of representative qPCR for each *EjLRR-RLK* genes.
