# Supplementary figures and images for: Genomic Survey of LRR-RLK Genes in Eriobotrya japonica and Their Expression Patterns Responding to Environmental Stresses
Source: Plants (Basel). 2024 Aug 27;13(17):2387. doi: 10.3390/plants13172387 (PMC11397332; doi:10.3390/plants13172387)

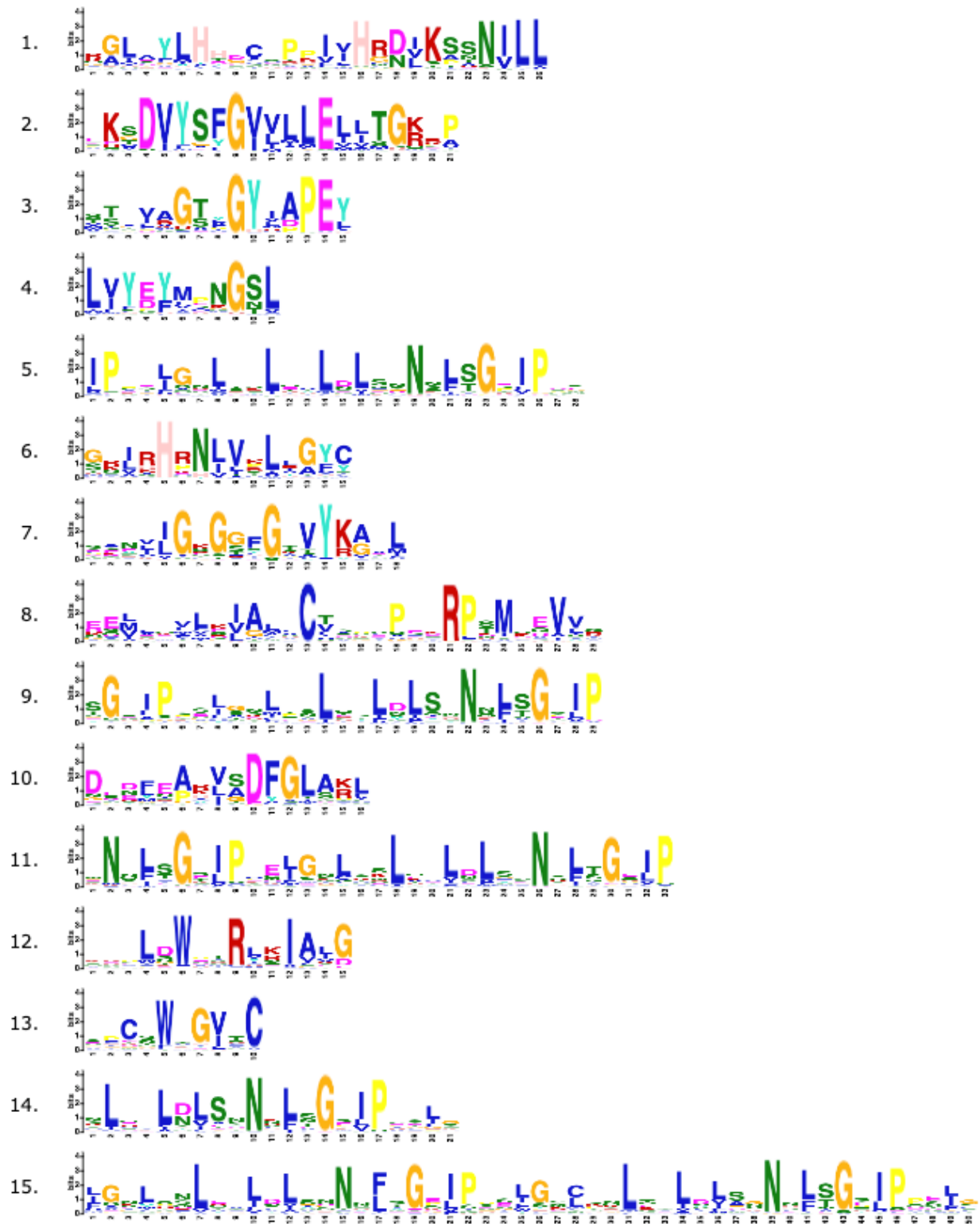

Supplementary Figure S3. 15 Motif logos of EjLRR-RLKs searched through the MEME tool.

Supplement: Supplementary file 1 [file plants-13-02387-s001.zip › Supplementary Figures/Supplementary Figure S3.pdf]
