# Supplementary material for: Genomic Survey of LRR-RLK Genes in Eriobotrya japonica and Their Expression Patterns Responding to Environmental Stresses
Source: Plants (Basel). 2024 Aug 27;13(17):2387. doi: 10.3390/plants13172387 (PMC11397332; doi:10.3390/plants13172387)

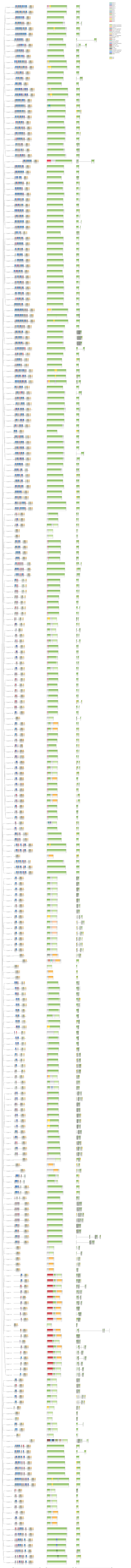

Supplement: Supplementary file 1 [file Supplementary Figure S1.pdf › Supplementary Figures/Supplementary Figure S1.pdf]
