# Supplementary material for: Genomic Survey of LRR-RLK Genes in Eriobotrya japonica and Their Expression Patterns Responding to Environmental Stresses
Source: Plants (Basel). 2024 Aug 27;13(17):2387. doi: 10.3390/plants13172387 (PMC11397332; doi:10.3390/plants13172387)
Supplement: Supplementary file 1 [file Supplementary Figure S2.pdf › Supplementary Figures/Supplementary Figure S2.pdf]

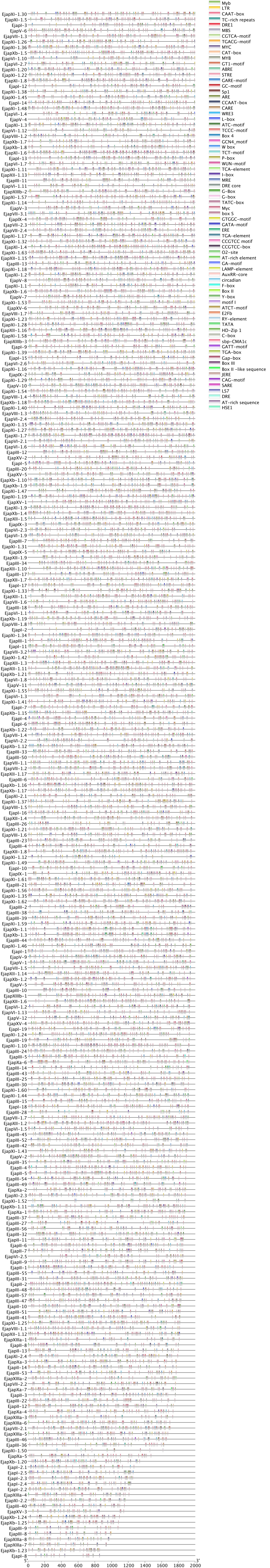
Myb
LTR
CAAT-box
TC-rich repeats
DRE1
MBS
CGTCA-motif
TGACG-motif
MYC
CAT-box
MYB
GT1-motif
ABRE
STRE
GARE-motif
GC-motif
Sp1
ARE
CCAAT-box
CARE
WRE3
L-box
ATC-motif
TCCC-motif
Box 4
GCN4_motif
W box
TCT-motif
P-box
WUN-motif
TCA-element
I-box
MRE
DRE core
G-Box
G-box
TATC-box
Myc
box S
GTGGC-motif
GATA-motif
ERE
TGA-element
CCGTCC motif
CCGTCC-box
O2-site
AT-rich element
GA-motif
LAMP-element
AuxRR-core
circadian
F-box
Box II
Y-box
motif I
ATCT-motif
E2Fb
RY-element
TATA
HD-Zip 1
C-box
sbp-CMA1c
GATT-motif
TGA-box
Gap-box
Box III
Box II -like sequence
JERE
CAG-motif
SARE
LS7
DRE
AT-rich sequence
HSE1
5'
3'
0
200
400
600
800
1000
1200
1400
1600
1800
2000
